# Catechins in stroke: multi-target mechanisms, preclinical evidence, and translational challenges

**DOI:** 10.3389/fphar.2026.1937660

**Published:** 2026-09-16

**Authors:** Yue Zhang, Shuangcui Wang, Qingqiang Yuan

**Affiliations:** 1 Institute of Rehabilitation Medicine, Qilu Medical University, Zibo, Shandong, China; 2 Department of Orthopedic Surgery, Hebei Medical University Third Hospital, Shijiazhuang, Hebei, China

**Keywords:** catechins, hemorrhagic stroke, ischemic stroke, neuroprotection, translational challenges

## Abstract

**Backgrounds:**

Stroke is a leading cause of death and disability, with limited therapeutic options available. Catechin is a natural polyphenolic compound that exhibits significant neuroprotective effects.

**Objectives:**

This review aims to summarize the pharmacokinetics of catechins, their neuroprotective mechanisms, preclinical evidence, and the translational challenges associated with both ischemic and hemorrhagic stroke.

**Methods:**

A structured literature search was conducted from 1985 to 2026 in PubMed, Web of Science, and Scopus, utilizing keyword combinations related to catechins and stroke.

**Results:**

Current preclinical research findings indicate that catechins can alleviate oxidative inflammation, modulate microglial polarization, inhibit apoptosis, and restore autophagic function. Additionally, they can maintain blood-brain barrier integrity and mitochondrial function, as well as promote angiogenesis and neurogenesis. These protective effects have been validated in experimental models of ischemic stroke, intracerebral hemorrhage, and subarachnoid hemorrhage. However, the existing mechanistic evidence regarding their neuroprotective effects is predominantly derived from studies on (−)-epigallocatechin-3-gallate (EGCG), while research on other catechin monomers, green tea extracts, or mixed polyphenol preparations remains relatively scarce.

**Conclusion:**

Catechins represent promising candidate drugs for stroke due to their multiple neuroprotective potentials. However, most preclinical evidence arises from pretreatment paradigms, which are confounded by heterogeneity in dosage, age, and experimental models. Additionally, the low bioavailability and narrow therapeutic window of catechins restrict their clinical translation. Future research should prioritize the development of nano-delivery systems, the optimization of dosing regimens, and the validation of findings in aged and comorbid models.

## 1 Introduction

The latest Global Burden of Disease 2021 report indicates that stroke emerged as the third leading cause of death worldwide in 2021, surpassed only by ischemic heart disease and COVID-19 (2024). As the primary contributor to adult disability, stroke imposes a substantial burden on both individuals and public health systems (Tsao et al., 2022).

This disease is characterized by acute, focal damage to the central nervous system resulting from vascular factors that disrupt normal cerebral circulation. It is classified into two primary categories: ischemic stroke and hemorrhagic stroke. Ischemic stroke is typically precipitated by the occlusion of cerebral arteries due to emboli or local vascular stenosis, leading to impaired cerebral blood flow. This impairment initiates a cascade of molecular events that ultimately result in neuronal injury and death. The depletion of oxygen and glucose leads to ATP depletion and the failure of Na^+^/K^+^-ATPase pumps, which causes sodium accumulation, membrane depolarization, and excessive release of glutamate (Hénaut et al., 2019; Qin et al., 2022). Overstimulation of N-methyl-D-aspartate (NMDA) receptors by glutamate triggers calcium influx and activates calcium-dependent degradative enzymes (Nakamura and Shichita, 2019). Calcium overload in the mitochondria opens the mitochondrial permeability transition pore (mPTP), dissipates the membrane potential, releases cytochrome c, and activates caspase-9 and caspase-3, thereby initiating intrinsic apoptosis (Gasull and Arboix, 2022; Qin et al., 2022). Damaged mitochondria produce excessive reactive oxygen species (ROS), leading to lipid peroxidation, protein oxidation, DNA damage, and the activation of nuclear factor ΚB (NF-κB) and hypoxia-inducible factor 1-alpha (HIF-1α) (Qin et al., 2022). NF-κB subsequently upregulates pro-inflammatory cytokines and inducible nitric oxide synthase (iNOS), exacerbating neuroinflammation (Hénaut et al., 2019; Qin et al., 2022).

Although the incidence of hemorrhagic stroke is lower than that of ischemic stroke, its associated disability and mortality rates are significantly higher (Rincon and Mayer, 2013). This condition arises from blood leakage into the brain parenchyma (intracerebral hemorrhage, ICH) or the subarachnoid space (subarachnoid hemorrhage, SAH) due to vascular rupture. Common etiological factors include hypertension, cerebrovascular structural abnormalities, anticoagulant or thrombolytic medications, and head trauma. Primary injury is a result of the mass effect of hematomas, while secondary injury is driven by hemoglobin-derived iron, which catalyzes Fenton reactions to produce hydroxyl radicals, along with thrombin and complement activation (Nakamura and Shichita, 2019). In both subtypes, matrix metalloproteinase-2 (MMP-2) and matrix metalloproteinase-9 (MMP-9) degrade tight junction proteins and components of the basement membrane, thereby disrupting the blood-brain barrier and promoting leukocyte infiltration and edema (Nakamura and Shichita, 2019; Qin et al., 2022). Additionally, ferroptosis contributes to secondary injury following ICH (Shehjar et al., 2023; Chen et al., 2026). Mitochondrial dysfunction, oxidative stress, and inflammation are interconnected processes that form the core of stroke pathophysiology and present multiple therapeutic targets (Lo et al., 2003; Qin et al., 2022).

Currently, the treatment of ischemic stroke primarily focuses on vascular recanalization and restoration of blood flow. Tissue plasminogen activator is the only thrombolytic drug approved by the U.S. Food and Drug Administration for acute ischemic stroke (Karaszewski et al., 2021). However, due to its narrow therapeutic time window and numerous contraindications, only a limited proportion of patients can actually receive this treatment (Endres et al., 2008). In contrast, the primary focus in hemorrhagic stroke is to control bleeding and reduce intracranial pressure. Current treatment recommendations mainly involve supportive measures, as there remains a lack of specific therapeutic drugs. Therefore, the development of effective alternative medications for stroke treatment is particularly urgent. In recent years, natural bioactive components have emerged as an ideal choice to complement or replace existing standard therapies due to their multi-target effects and low toxicity. Studies have indicated that catechins, which are lipophilic and multi-target, exhibit potential value in the prevention and treatment of stroke. However, the safety profile of catechins is dose-dependent rather than inherently non-toxic (Hu et al., 2018). At low to moderate doses consistent with dietary intake (e.g., 90–300 mg (−)-epigallocatechin-3-gallate (EGCG)/day from green tea infusions), catechins are generally recognized as safe and exert beneficial antioxidant effects (Younes et al., 2018). In contrast, high-dose supplementation (typically ≥800 mg EGCG/day) has been associated with hepatotoxicity in susceptible individuals (Hu et al., 2018; Ferrari and Naponelli, 2025). Clinical trials have reported elevated serum transaminases in subjects consuming high-dose green tea extracts, with cases of severe liver injury documented at doses exceeding 800 mg EGCG/day, particularly when administered as a bolus on an empty stomach (Younes et al., 2018; Acosta et al., 2023). The pro-oxidant activity of catechins at high concentrations, whereby EGCG transitions from radical scavenger to ROS generator, underpins this dose-dependent toxicity (Ouyang et al., 2020). Therefore, while catechins demonstrate neuroprotective potential against stroke through multi-target mechanisms, their therapeutic application requires careful dose optimization and consideration of individual susceptibility factors.

Catechins are naturally occurring polyphenolic compounds found in various plant-based foods and medicinal herbs. Their primary dietary source is green tea (Camellia sinensis), which is particularly rich in EGCG (−)-epigallocatechin (EGC) (−)-epicatechin gallate (ECG), and (−)-epicatechin (EC) (Nagao et al., 2007; Xu et al., 2017; Pervin et al., 2019; Meyer et al., 2023). Catechins are also abundant in fruits such as grapes, apples, berries, and stone fruits, as well as in cocoa beans, legumes, and nuts (Pervin et al., 2019; Meyer et al., 2023). Furthermore, catechins are present in numerous traditional Chinese medicine (TCM), including *Polygonum multiflorum* (Han et al., 2013; Liu et al., 2020), *Rheum palmatum* (Xiong et al., 2023; Yang et al., 2024), among others. Existing research indicates that catechins possess extensive medicinal properties, including antioxidant (Meyer et al., 2023), anti-aging (Si et al., 2021), antiviral (Xu et al., 2017), anticancer (Farhan, 2022), hypoglycemic (Bakhtiyari et al., 2019), hypolipidemic (Tang et al., 2023), immunomodulatory (Andrade et al., 2024), pain-relieving (Quiñonez-Bastidas et al., 2018), neuroprotective (Nehlig, 2013), and cerebral ischemic injury-prevention effects (Nichols et al., 2015). Studies have also shown that the intake of catechin-rich foods has significant positive effects on health, including a reduction in the risks of cardiovascular diseases, diabetes, and obesity (Nagao et al., 2007). Interestingly, catechins are beneficial for both ischemic and hemorrhagic strokes. This review will elucidate the specific molecular mechanisms underlying the multi-target neuroprotective effects of catechins in stroke, summarize evidence from various disease models, and explore the prospects and challenges of clinical translation. Before dissecting the molecular mechanisms, we first summarize the chemical properties and pharmacokinetic profiles of catechins, as these directly influence their bioactivity and brain penetration.

## 2 Methods

This narrative review summarizes and critically evaluates the literature on the pharmacokinetics, neuroprotective mechanisms, preclinical evidence, and translational challenges of catechins in stroke. A structured literature search was performed on 1 June 2026 in PubMed, Web of Science, and Scopus. Full-text searches were conducted in PubMed and Web of Science using keyword combinations related to catechins (catechin OR epigallocatechin-3-gallate OR epicatechin OR gallocatechin OR epigallocatechin OR catechin gallate OR epicatechin gallate OR gallocatechin gallate) and stroke (stroke OR ischemic stroke OR hemorrhagic stroke OR neuroprotection). For Scopus, which yielded a substantially larger initial result set, the search was refined to title, abstract, and keyword fields using the same keyword combinations to improve specificity while maintaining adequate sensitivity.

Studies were considered for inclusion if they were peer-reviewed original articles or reviews relevant to the pharmacokinetics, pharmacological mechanisms, preclinical effects, or translational aspects of catechins in stroke. Exclusion criteria were: (i) duplicate publications; (ii) studies irrelevant to the neuroprotective effects of catechins or stroke; (iii) studies with major methodological limitations (e.g., absence of appropriate controls, unclear sample size, incomplete statistical analyses); (iv) conference abstracts, case reports, and editorials.

Two independent reviewers screened titles and abstracts, followed by full-text assessment against the predefined criteria. Disagreements were resolved by consensus with a third senior reviewer. The literature selection process for all references cited in this review is illustrated in a Preferred Reporting Items for Systematic Reviews and Meta-Analyses (PRISMA) flow diagram (Supplementary Figure S1). Through this process, a total of 208 references were deemed eligible and were cited in this review.

## 3 Chemical properties and pharmacokinetic characteristics of catechin

### 3.1 Structural basis for catechin bioactivity and metabolism

The catechin monomers primarily include (+)-catechin, EC (+)-gallocatechin (GC), EGC (+)-catechin gallate (CG), ECG (+)-gallocatechin gallate (GCG), and EGCG (Hirao et al., 2010; Braicu et al., 2013) (Figure 1). Their basic structure contains two benzene rings (A ring and B ring) and a dihydropyran heterocyclic ring (C ring). A hydroxyl group is attached to the C3 position, and C2 and C3 act as two chiral centers (Bansal et al., 2013). In certain catechins, such as ECG and EGCG, the hydroxyl group at carbon 3 of the C ring is esterified with gallic acid, introducing a gallate ester group that forms the D ring (Bansal et al., 2013).

**FIGURE 1:**
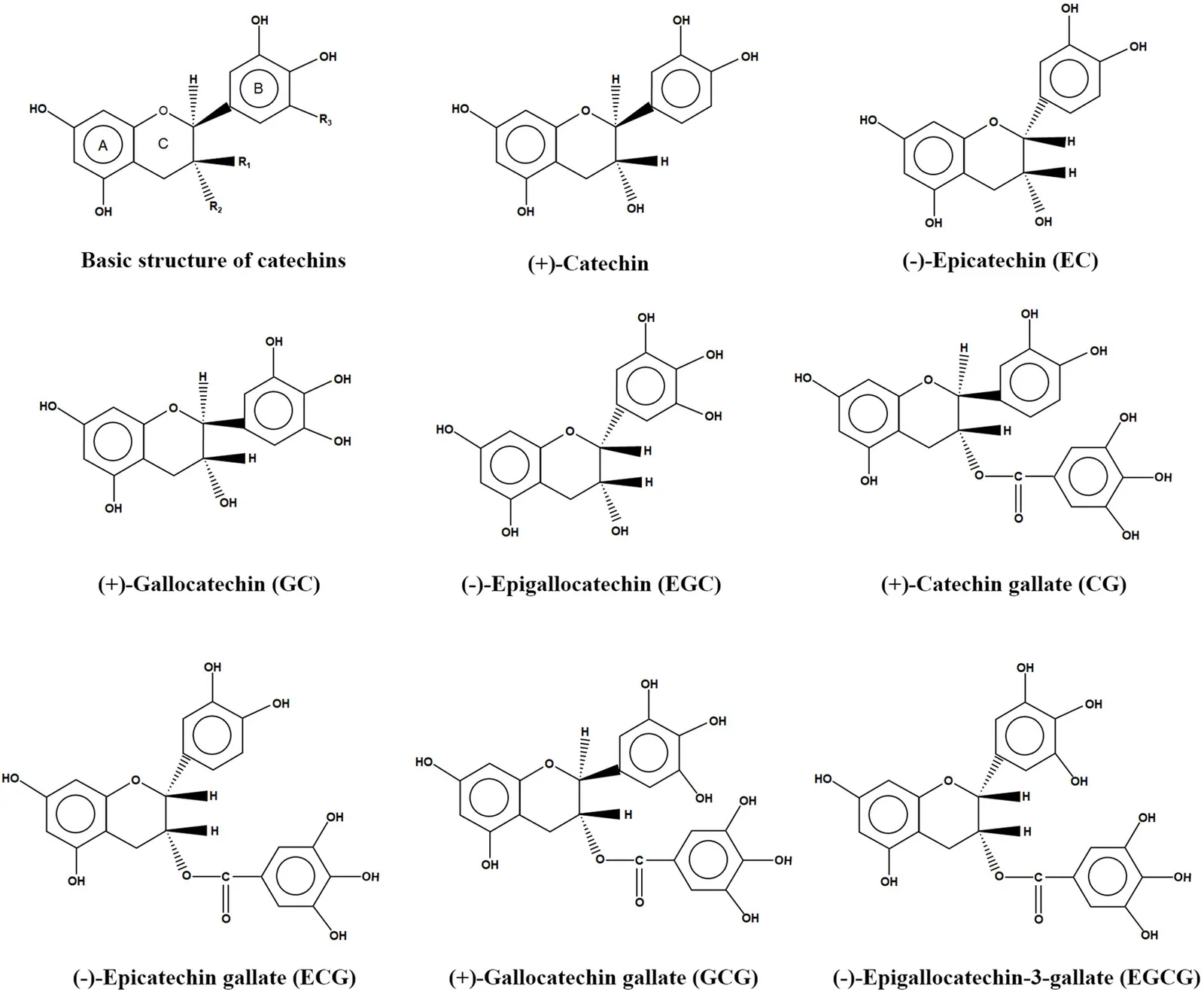
The chemical structures of major catechin derivatives.

The bioactivity of catechins is closely linked to their structural characteristics. Most catechins feature either two (3′,4′) or three (3′,4′,5′) phenolic hydroxyl groups on the B ring. Studies have shown that a B ring with three phenolic hydroxyl groups is more susceptible to oxidation than one with only two. These hydroxyl groups exhibit a strong hydrogen-donating capacity and can form stable intramolecular hydrogen bonds with adjacent hydroxyl groups, thereby enhancing their free radical scavenging effects. In comparison to the catechol structure, which contains two hydroxyl groups, the pyrogallol structure with three hydroxyl groups demonstrates a stronger reducing capacity due to its higher phenolic hydroxyl content (Wang et al., 2023). Moreover, the galloyl group on the D ring plays a crucial role in the antioxidant activity of catechins. Catechins containing a D ring (such as CG, ECG, GCG, EGCG) exhibit significantly stronger antioxidant effects compared to their corresponding monomers lacking a D-ring (such as C, EC, GC, EGC) (Wang et al., 2023). Reports indicate that the oxidative degradation tendency of tea catechins follows the order EGCG > EGC > ECG > EC. The hydroxyl groups at the 5- and 7-positions of the A ring and at the 3-position of the C ring often undergo glycosylation modifications, which help protect the active hydroxyl groups from auto-oxidation (Xiao, 2017). However, their hydrogen-donating capacity is notably weaker than that of the hydroxyl groups on the B ring and D ring. The resorcinol structure on the A ring itself exhibits relatively low antioxidant activity, but it can synergistically enhance the hydrogen-donating capacity of phenolic hydroxyl groups on other rings (Krych and Gebicka, 2013).

Catechins can be metabolized *in vivo* into various secondary products, including glucuronide conjugates, sulfate esters, methylated metabolites, quinone derivatives, as well as phenolic acids and their glycine conjugates. Among these, the phenolic acids and their glycine conjugates are primarily produced by the action of intestinal microbiota (Feng, 2006; Muzolf-Panek et al., 2008; Fraga and Oteiza, 2011; Brückner et al., 2012; Clifford et al., 2013). These metabolic transformations can significantly influence their biological effects. Intracellular EGCG-3′-glucuronide can be converted into free EGCG through deglycosylation, thereby enhancing its bioavailability (Brückner et al., 2012). Therefore, maintaining the structural stability of catechins is crucial for their biochemical functions.

### 3.2 Absorption, distribution, metabolism and excretion

The bioavailability of catechins in the human body is relatively low (Ezzat et al., 2019). Studies have demonstrated that less than 5% of the orally administered catechin dose enters systemic circulation in rats (Lin et al., 2007). In humans, approximately 1.68% of ingested catechins are distributed as 0.16% in plasma, 1.1% in urine, and 0.42% in feces 6 hours after oral administration (Warden et al., 2001). Pharmacokinetic studies further indicate that detectable levels in blood are approximately 14% of the administered dose for EGC, 31% for EC, and less than 1% for EGCG (Chen et al., 1997).

Catechins are primarily absorbed in the gastrointestinal tract (Fan et al., 2017). Their absorption and metabolism involve multiple processes, including initial modification in the acidic gastric environment, uptake by the small intestinal mucosa, enterohepatic circulation, and metabolic transformation by colonic microbiota (Clifford et al., 2013; Stevens and Maier, 2016). Only a small fraction of orally ingested catechins is absorbed in the small intestine, subsequently undergoing phase I (hydrolysis) and phase II (methylation, glucuronidation, and sulfation) metabolism in enterocytes and hepatocytes before entering systemic circulation (Lambert et al., 2007).

After entering circulation, catechins are primarily distributed and eliminated through several pathways, including distribution via blood to various organs and tissues to exert their pharmacological effects, filtration by the kidneys and subsequent excretion in urine, and excretion into the intestinal tract via bile, which may re-enter the body through enterohepatic circulation. Notably, approximately 60%–70% of catechins undergo microbial degradation in the colon, including those that are excreted into the intestinal tract via bile.

EC demonstrates the highest bioavailability among various catechins, with approximately 20%–30% of the ingested amount being directly absorbed into the bloodstream through the small intestine (Liu et al., 2024). Even when administered via intravenous injection, catechins undergo partial degradation before reaching their target tissues (Li et al., 2012). Additionally, intestinal epithelial cells lack specific transport proteins for catechins, so their trans-epithelial transport primarily relies on passive diffusion driven by concentration gradients (Cai et al., 2018).

### 3.3 Blood-brain barrie (BBB) permeability

The BBB is a multicellular structural barrier that separates blood from brain tissue, primarily functioning to strictly regulate substances entering the brain, including blocking metabolic waste and harmful substances present in the blood. However, this barrier also presents a significant challenge for most drugs attempting to reach brain tissue, particularly large-molecule, water-soluble, or charged drugs that often struggle to penetrate it. Consequently, many therapeutic drugs intended to treat brain diseases fail to effectively reach their target sites.

The penetration of catechins across the BBB has been investigated through various methods, including oral administration, intravenous injection, and *in vitro* models. These approaches vary in their ability to confirm brain entry or establish pharmacologically active concentrations.

Initial evidence for catechin penetration into the brain was derived from studies involving oral administration. Inanami et al. reported that oral administration of (−)-catechin reduced neuronal death induced by ischemia/reperfusion (I/R) in gerbils; however, brain concentrations were not measured (Inanami et al., 1998). Conversely, Pervin et al. detected orally administered EGCG in mouse brain at a concentration of 0.14 nmol/g (Pervin et al., 2017). Which is below the required range of 1–100 µM for neuroprotection *in vitro* (Fu and Koo, 2006; Jung et al., 2007b; Srividhya and Kalaiselvi, 2013).

Intravenous administration studies provide more definitive evidence of BBB permeability by bypassing the barriers of oral absorption. Using microdialysis sampling coupled with high-performance liquid chromatography, Liang et al. identified (+)-catechin and EC in both rat brain tissue and blood after intravenous injection, confirming that these compounds can cross the BBB when delivered directly into the circulation (Liang et al., 2014). Complementary evidence from *in vitro* BBB models has further elucidated the transport mechanisms. Faria et al. demonstrated apical-to-basolateral transport of (+)-catechin and EC across immortalized rat brain capillary endothelial and human cerebral microvascular endothelial monolayers, confirming the capacity of these compounds to traverse an endothelial barrier (Faria et al., 2011).

Collectively, these findings establish that catechins possess intrinsic BBB permeability. However, the concentrations achieved in the brain after oral administration are typically in the nanomolar range (Nakagawa and Miyazawa, 1997). These values are orders of magnitude lower than those required for direct neuroprotective activity *in vitro* (Reznichenko et al., 2005). This concentration gap suggests that the beneficial effects of orally administered catechins in stroke models may be mediated through multiple, potentially overlapping mechanisms: microbial ring-fission metabolites with greater BBB permeability (Pervin et al., 2019), indirect peripheral actions such as modulation of systemic inflammation or oxidative stress, or accumulation in specific brain regions at concentrations not reflected in whole-tissue homogenates (Lin et al., 2007). Future studies employing quantitative microdialysis or brain region-specific analyses will be essential to establish the relationship between catechin concentrations at relevant targets and functional neuroprotective outcomes (Lin et al., 2007).

## 4 Mechanisms of catechin in protecting against stroke

Catechins are structurally poised to interfere with multiple stroke-related pathological cascades. However, most mechanistic evidence derives from EGCG, with limited data for other catechins or mixed polyphenol preparations. The following sections dissect these multi-target mechanisms.

### 4.1 Suppressing the oxidative-inflammatory network

Stroke induces a self-amplifying vicious cycle between oxidative stress and neuroinflammation, which drives secondary brain damage. Excessive ROS activate transcription factors including NF-κB, thereby promoting the release of pro-inflammatory cytokines; in turn, inflammatory mediators further boost ROS production from mitochondria and NADPH oxidases. Catechins interact with this network through various mechanisms, including direct radical scavenging, activation of endogenous antioxidant defenses, and modulation of microglial phenotypes. Notably, the evidence supporting these effects primarily stems from studies focused on EGCG, while comparatively limited data exists for other monomers.

#### 4.1.1 Activating the endogenous antioxidant defense

EGCG is the most extensively studied catechin for its antioxidant properties. In stroke-prone spontaneously hypertensive rats, EGCG has been shown to prevent the occurrence of strokes by eliminating free radicals and inhibiting lipid peroxidation (Uchida et al., 1995). In PC12 cells, EGCG effectively reversed H_2_O_2_-induced oxidative damage, reduced protein carbonylation, restored thiol status, and maintained mitochondrial membrane potential (Srividhya and Kalaiselvi, 2013). Further studies have confirmed that EGCG intervention can prevent neuronal damage in rats with cerebral ischemia by scavenging peroxynitrite through its antioxidant actions (Nagai et al., 2002). Mechanistically, EGCG activates the nuclear factor erythroid 2-related factor 2 (Nrf2)/Antioxidant Response Element (ARE) pathway following focal cerebral ischemia (Han et al., 2014). However, the effects of EGCG are dose-dependent. In glutamate-treated HT-22 cells, EGCG at a concentration of 1 µM reduced ROS, whereas concentrations of 100 µM and above increased heme oxygenase 1 (HO-1) mRNA expression, suggesting that high doses may induce cellular stress that paradoxically upregulates protective enzymes (Fu and Koo, 2006). Notably, the protective mechanism of EGCG is dual, in that it can activate the Nrf2-dependent pathway (such as upregulating phase II enzymes) while also preventing ICH injury through independent pathways, such as suppressing activator protein-1 (AP-1) activity and reducing ferroptosis-related gene expression (Chang et al., 2014). Beyond ischemic and hemorrhagic stroke models, both *in vitro* and *in vivo* experiments confirmed that EGCG can reduce malondialdehyde (MDA) production and neuronal damage induced by excitotoxins such as NMDA and α-amino-3-hydroxy-5-methyl-4-isoxazolepropionic acid (AMPA) (Lee et al., 2004).

EC has also been shown to activate the Nrf2/HO-1 pathway as a key mechanism for its antioxidant effects. EC promotes Nrf2 nuclear translocation, enhancing HO-1 expression in a dose-dependent manner (Shah et al., 2010). This protection has been validated in aged ischemic stroke models and ICH models. In aged rats subjected to middle cerebral artery occlusion/reperfusion (MCAO/R) and senescent SH-SY5Y cells exposed to oxygen-glucose deprivation/reoxygenation (OGD/R), EC alleviated injury by upregulating Nrf2 (Jiang et al., 2024). Building on these findings, Lan et al. further explored the underlying mechanisms. By treating primary astrocytes derived from wild-type and Nrf2 knockout mice with hemoglobin to establish an *in vitro* ICH model, researchers found that EC protects cells from hemoglobin toxicity through a dual mechanism. One pathway involves Nrf2 upregulation, while the other suppresses AP-1 activity via an Nrf2-independent mechanism (Lan et al., 2017).

Other individual catechins have received limited attention regarding their role in antioxidant defense mechanisms. In rat astrocytes, treatment with catechin significantly enhanced superoxide dismutase (SOD) activity, exhibiting an effect that was even more pronounced than that of vitamin E (Chan et al., 2002). Nevertheless, systematic studies investigating ECG, EGC, GC, or GCG as individual agents within stroke-related oxidative stress models are notably scarce, and the existing evidence is insufficient to ascertain whether these monomers possess the Nrf2-activating properties characteristic of EGCG and EC.

#### 4.1.2 Modulating microglial activation and inflammatory pathways

EGCG is the most extensively studied catechin regarding its anti-inflammatory effects in stroke models. In rats subjected to cerebral I/R, immediate treatment with EGCG resulted in a significant reduction of tumor necrosis factor-α (TNF-α), interleukin-1 beta (IL-1β), and interleukin-6 (IL-6) levels. Furthermore, EGCG inhibited the upregulation of NF-κB/nuclear factor kappa-light-chain-enhancer of activated B cells p65 subunit (p65) and decreased the expression of cyclooxygenase-2 (COX-2) and iNOS (Zhang et al., 2015). These findings were confirmed in middle cerebral artery occlusion (MCAO) and OGD/R models, where EGCG also upregulated the anti-inflammatory cytokine IL-10 (He et al., 2021).

Mechanistically, EGCG inhibits the activation of signal transducer and activator of transcription 1 (STAT1), which is a key driver of iNOS-mediated NO production (Mariotto et al., 2004; Darra et al., 2007). Additionally, the multi-target regulation of the NF-κB pathway by EGCG has garnered significant attention. Zhong et al. employed lipopolysaccharide (LPS) and amyloid beta (Aβ)-induced microglia *in vitro*, as well as amyloid precursor protein (APP)/presenilin 1 (PS1) mice *in vivo*, to demonstrate that EGCG exerts its effects, at least partially, by inhibiting the toll-like receptor 4 (TLR4)/NF-κB pathway (Zhong et al., 2019). In CoCl_2_-induced hypoxic BV-2 microglial cells, EGCG effectively reduced the levels of IL-6, iNOS, and COX-2 by blocking NF-κB and HIF-1α (Kim et al., 2022).

EGCG also modulates the phenotype and function of microglia, facilitating their transition from a pro-inflammatory (M1) state to a reparative (M2) state, as evidenced by increased expression of cluster of differentiation 206 (CD206) (Machin et al., 2022). This transition enhances neurogenesis and promotes functional recovery following stroke (Shin et al., 2014; Zhang et al., 2017). Additionally, EGCG protects neurons from damage by inhibiting the secretion of neurotoxic inflammatory mediators from microglia. Voltage-gated proton channels, which are expressed in microglia, are essential for NADPH oxidase-dependent ROS generation. The extent of brain injury following ischemic stroke relies on proton channel activity, and EGCG effectively inhibits proton currents in BV-2 microglia without altering the gating process of these channels. This finding suggests that the inhibition of proton channels may be a key mechanism through which EGCG suppresses microglial activation and subsequent neurotoxic events (Jin et al., 2013). Neuronal damage or neuroinflammation can lead to the activation of microglia, which subsequently induces the production of NO. NO can interact with key cysteine thiols of target proteins, resulting in the formation of S-nitrosoproteins. This post-translational modification, known as S-nitrosylation, plays a critical role in inflammatory signal transduction. Qu et al. exposed immortalized mouse BV-2 microglial cells to a physiological NO donor or to endotoxin LPS *in vitro* to induce pro-inflammatory responses. Through this model, they demonstrated that EGCG could downregulate the levels of protein S-nitrosylation in LPS-stimulated BV-2 microglial cells, which aligns with its established antioxidant effects (Qu et al., 2014).

Catechin hydrate has also been investigated, although to a lesser extent. In wistar rats pretreated with catechin hydrate for 21 days prior to MCAO, animals exhibited smaller infarcts, reduced neurological deficits, and lower expression of iNOS, glial fibrillary acidic protein (GFAP), and NF-κB (Ashafaq et al., 2012). However, this study employed a pretreatment regimen rather than post-stroke therapeutic administration, limiting its direct translational relevance and reflecting a preconditioning effect rather than therapeutic efficacy after stroke onset.

In summary, the anti-inflammatory effects of catechins, particularly the inhibition of NF-κB, STAT1, and TLR4 pathways, as well as the promotion of M2 microglial polarization, are primarily supported by EGCG studies (Figure 2). Although the antioxidant effects have been consistently reported across various studies, the translation of these findings is complicated by the dose-dependent pro-oxidant activities observed at high concentrations and the lack of standardized dosing protocols across different experimental models. Furthermore, the relative contributions of Nrf2-dependent and Nrf2-independent pathways to catechin-mediated protection remain ambiguous, as most studies use pharmacological inhibitors rather than genetic knockout approaches.

**FIGURE 2:**
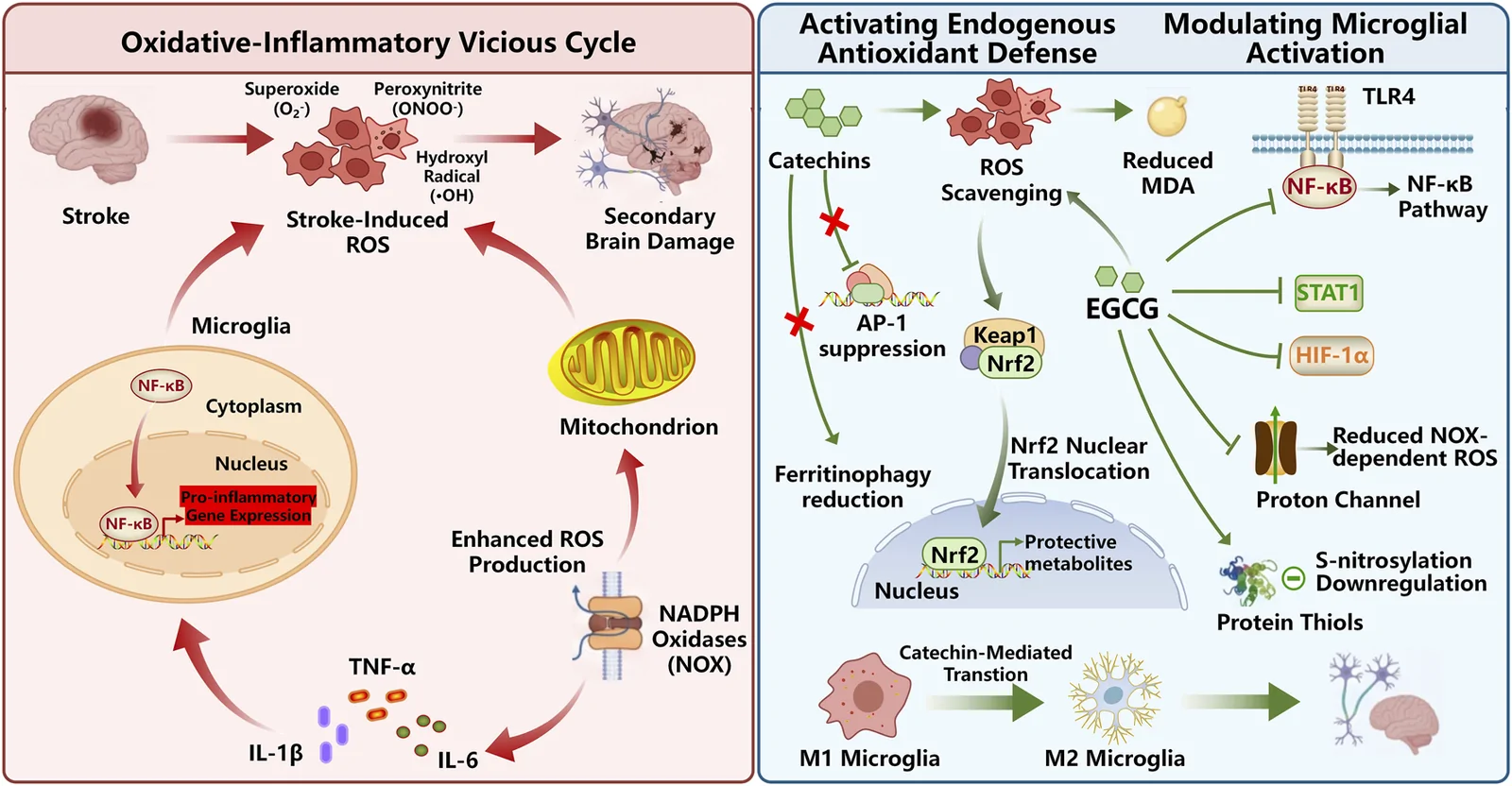
Catechins effectively suppress the oxidative-inflammatory network following a stroke. They inhibit the production of ROS and the expression of pro-inflammatory genes mediated by NF-κB. Concurrently, catechins enhance antioxidant defenses and modulate microglial polarization, transitioning from a pro-inflammatory (M1) phenotype to an anti-inflammatory (M2) phenotype, thereby mitigating secondary brain injury.

### 4.2 Blocking programmed cell death: anti-apoptosis and autophagy regulation

Oxidative stress and inflammation converge on cellular death pathways, including apoptosis, necroptosis, and autophagic cell death. Catechins intervene at multiple nodes to promote survival.

#### 4.2.1 Mitochondria-dependent and -independent anti-apoptosis

EGCG is the most extensively studied catechin for its anti-apoptotic effects. Catechins not only demonstrate preventive effects but also possess therapeutic potential for intervention following injury. In a ‘neurorescue’ model simulating clinical treatment windows, the administration of EGCG after the onset of serum deprivation-induced PC12 cell death significantly reversed cell death. This salvage effect is dependent on the activation of the protein kinase C (PKC) signaling pathway (Reznichenko et al., 2005). At the molecular level, EGCG has also been shown to regulate the thioredoxin system, preserving its interaction with apoptosis signal-regulating kinase 1 (ASK1) and preventing oxidative stress-induced apoptosis (Park et al., 2021b).

EGCG regulates the B-cell lymphoma 2 (Bcl-2) family to inhibit mitochondrial-mediated apoptosis. In PC12 cells exposed to the NO donor sodium nitroprusside (SNP), EGCG improved the Bcl-2 associated X protein (Bax)/Bcl-2 ratio, reduced caspase-9, -8, and -3 activities, prevented cytochrome c release, and downregulated the expression of the voltage-dependent anion channel (VDAC) (Jung et al., 2007a). Similar effects were observed in CoCl_2_-induced hypoxic PC12 cells, where EGCG restored mitochondrial membrane potential (ΔΨm), inhibited DNA fragmentation, and reversed the increased Bax/Bcl-2 ratio (Jung et al., 2007b). In the MCAO rat model, EGCG reduced caspase-3 and poly (ADP-ribose) polymerase (PARP) expression, exerting neuroprotective effects through modulation of the apoptotic signaling cascade (Park et al., 2020). Furthermore, EGCG was confirmed to reduce the number of necrotic neurons and ameliorate the morphological changes associated with necroptosis in MCAO model rats (Machin et al., 2021).

The phosphoinositide 3-kinase (PI3K)/Akt pathway is a key pro-survival mediator. Upon activation, PI3K triggers Akt, which subsequently inactivates Bax, thereby preventing the formation of Bax/Bcl-2 heterodimers and allowing Bcl-2 to exert its anti-apoptotic effects. EGCG intervention in MCAO rats reduced apoptosis, upregulated Bcl-2, and downregulated caspase-3 and Bax, effects linked to PI3K/Akt/eNOS signaling (Nan et al., 2018). In a H_2_O_2_-induced oxidative damage model of PC12 cells, EGCG-mediated activation of PI3K/Akt and inhibition of glycogen synthase kinase-3 (GSK-3) have also been highlighted as a potential therapeutic strategy (Koh et al., 2003).

EGCG also contributes to anti-apoptosis through multiple additional mechanisms. These include upregulating hippocalcin to prevent calcium overload (Park et al., 2024) and inhibiting methylglyoxal (MGO)-induced glycation end product (AGE) formation and mitogen-activated protein kinase (MAPK)-dependent neurotoxicity (He et al., 2022). Furthermore, in MCAO models, EGCG suppresses endoplasmic reticulum stress markers, specifically glucose-regulated protein 78 (GRP78), C/EBP-homologous protein (CHOP), and caspase-12 (Yao et al., 2014).

In contrast, EC has been investigated to a much lesser extent. In the global ischemia models, EC reduces the pro-nerve growth factor (pro-NGF)/NGF ratio and activates tropomyosin kinase receptor (Trk) while suppressing Jun-kinase (JNK)-mediated signaling via p75 neurotrophin receptor (p75NTR) (Choucry et al., 2019).

Other individual catechins and mixed extracts have received limited attention regarding their anti-apoptotic properties. Utilizing the OGD/R model in PC12 cells, Gundimeda et al. demonstrated that pretreatment with green tea polyphenols protected cells from OGD/R-induced cell death. Further mechanistic studies confirmed that this protective effect was mediated by the stimulation of the 67-kDa laminin receptor (67LR), ROS production, and activation of PKCε. Notably, EGCG, the primary active component in green tea polyphenols, exhibited a high affinity for 67LR, indicating its significant role in eliciting this pharmacological effect (Gundimeda et al., 2012).

In summary, the anti-apoptotic effects of catechins, particularly those involving PKC/67LR signaling, regulation of the Bcl-2 family, activation of the PI3K/Akt pathway, maintenance of calcium homeostasis, and modulation of endoplasmic reticulum stress, are strongly supported by studies on EGCG. However, the anti-apoptotic potential of catechins as a class should be interpreted with caution, as the current evidence base is predominantly derived from EGCG.

#### 4.2.2 Balancing autophagy: from pro-survival to preventing autophagic death

Autophagy plays a dual role in stroke—protective at moderate levels but detrimental when excessive. Catechins restore autophagic homeostasis in a manner that is dependent on both cell type and context. However, the existing evidence mainly relies on static markers rather than dynamic flux assays.

EGCG has been the primary focus of autophagy research in stroke models. In the SAH model, a 7-day pre-administration regimen blocks ferrous iron accumulation via HO-1-associated iron-metabolism shifts, ameliorates oxidative stress and mitochondrial dysfunction, and is accompanied by attenuated ferroptosis and ferritinophagy markers (Huang et al., 2024). EGCG also modulates autophagy-related markers, including autophagy-related 5 (Atg5), microtubule-associated protein 1 light chain 3 beta (LC3B), and Beclin-1, suggesting an influence on autophagosome dynamics (Chen et al., 2017). However, these studies rested on static marker reads without lysosomal-blockade flux assays (chloroquine/bafilomycin A1), so enhanced flux vs. impaired degradation cannot be distinguished. Beyond SAH, EGCG inhibits ferroptosis in ICH models by activating Nrf2-Kelch-like ECH-associated protein 1 (Keap1), promoting Nrf2 nuclear translocation and HO-1 upregulation (Zhou et al., 2026). Collectively, EGCG modulates autophagic and iron-homeostatic activity, rather than definitively restoring it. Claims of a “balanced autophagic state” remain inferential, given the absence of direct flux measurements in the cited works.

Other catechins have received limited attention regarding their role in autophagy regulation in stroke. In hypoxia/reperfusion models (+)-catechin suppress detrimental autophagy triggered by excessive ROS in microglia by upregulating the PI3K/Akt/Mechanistic target of rapamycin Kinase (mTOR) signaling pathway, thereby maintaining cell viability and preventing autophagic death (Chen et al., 2016).

In summary, the regulation of autophagy by catechins in stroke models is primarily supported by EGCG studies, with limited data available for other monomers. Catechins block apoptosis via mitochondrial pathways (Bcl-2/caspase), PI3K/Akt signaling, calcium homeostasis, and MAPK modulation. Simultaneously, they appear to modulate autophagic activity, potentially preventing autophagic cell death while enabling clearance of damaged mitochondria. However, definitive conclusions about the restoration of autophagic flux await studies that use dynamic flux assays (Figure 3). Autophagy plays a dual role in stroke; moderate autophagy protects neural tissue, while excessive autophagy can lead to damage. The regulation of autophagy by catechin exhibits inconsistent effects across microglia, neurons, and various stroke models. This contextual variability implies that catechin treatment possesses a narrow and unpredictable therapeutic window. Moreover, the lack of dynamic flux measurements in most studies limits the ability to determine whether the observed changes in LC3B and other markers reflect enhanced autophagic activity, impaired degradation, or a combination of both.

**FIGURE 3:**
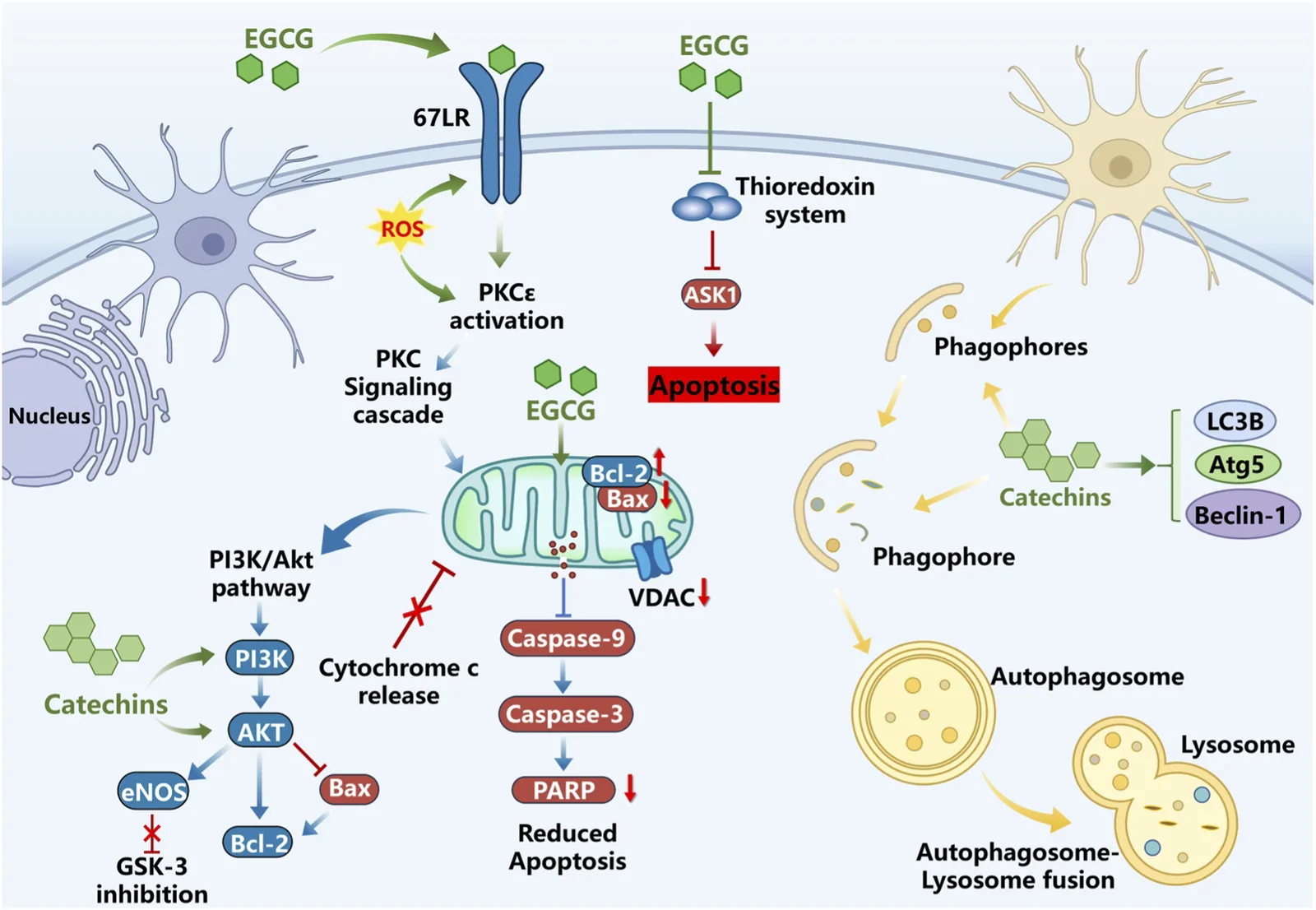
Catechins inhibit programmed cell death through anti-apoptotic mechanisms and the regulation of autophagy. Both catechins and EGCG activate the PI3K/Akt/mTOR signaling pathway, which results in the suppression of excessive autophagy and apoptosis. This process involves the inhibition of cytochrome c release and the modulation of Bax/Bcl-2-mediated mitochondrial dysfunction. Furthermore, catechins stimulate the thioredoxin system and facilitate the fusion of autophagosomes with lysosomes, thereby maintaining cellular homeostasis.

### 4.3 Preserving BBB integrity and mitochondrial function

BBB and mitochondria are two critical structures whose dysfunction exacerbates stroke pathology. Catechins protect both through overlapping mechanisms involving matrix metalloproteinases, tight junction proteins, and mitochondrial dynamics.

#### 4.3.1 Protecting BBB integrity via MMP-9 inhibition

The BBB, serving as the guardian and regulator of the central nervous system, effectively protects the brain from harmful substances under physiological conditions. However, during ischemic stroke and subsequent I/R injury, the BBB becomes disrupted (Fang et al., 2015). Oxidative stress, induced by free radical generation and metabolic imbalance, is a primary factor initiating BBB damage (Alfieri et al., 2013; Yu et al., 2014). The loss of BBB integrity not only promotes the formation of vasogenic brain edema but also accelerates the infiltration of circulating leukocytes, thereby exacerbating barrier disruption and cerebral inflammatory responses, creating a vicious cycle (Choi et al., 2011; Huang et al., 2013). MMP-9 is a crucial effector that degrades the extracellular matrix and cleaves tight junction proteins, including occludin and claudins.

EGCG has been shown to protect BBB integrity in stroke models. In a transient global ischemia model using C57BL/6 mice, immediate post-ischemic injection of EGCG markedly reduced MMP-9 activity and levels, consequently decreasing the incidence of delayed neuronal death (Park et al., 2009; Park et al., 2010). These findings indicate that EGCG’s protective effect on the BBB is mediated at least in part through inhibition of MMP-9.

Catechin has also been investigated for its effects on BBB integrity. In rats with cerebral I/R, catechin injection significantly alleviated BBB damage and interstitial edema (Fang et al., 2001).

Additionally, EC has been studied primarily in the context of age-dependent BBB protection. In 12-month-old mice with permanent cerebral ischemia, EC’s protective effects were independent of BBB preservation, suggesting that the glial responses seen in younger mice might represent compensatory mechanisms that are altered in the aging brain (Leonardo et al., 2013; Leonardo et al., 2015). BBB disruption and mitochondrial dysfunction often synergistically amplify secondary brain injury. Catechins simultaneously target both structures to stabilize the neurovascular unit.

In summary, the evidence for catechin-mediated BBB protection is derived from studies of EGCG, catechin, and EC, with substantial methodological heterogeneity across studies. EGCG has been most extensively characterized in the context of MMP-9 inhibition, while EC has been studied primarily in aged models with findings suggesting that BBB protection may not be its primary mechanism. The existing data on other catechin monomers are insufficient to draw meaningful conclusions, and it cannot be assumed that the effects of different catechins on BBB integrity are equivalent.

#### 4.3.2 Maintaining mitochondrial dynamics and function

Mitochondrial dysfunction is a critical contributor to stroke pathology, and catechins have been shown to protect these organelles through multiple mechanisms. Notably, the evidence for these effects is predominantly derived from EGCG, with some data available for EC and limited information for other monomers.

EGCG is the most extensively studied catechin for its mitochondrial protective effects. EGCG accumulates in mitochondria (Schroeder et al., 2009) and exerts direct effects on these organelles, improving mitochondrial function and regulating autophagic flux through a multi-target synergistic mechanism. EGCG markedly preserves the activity of mitochondrial respiratory chain complexes (I-V) and citrate synthase, demonstrating its capacity to sustain mitochondrial energy metabolism (oxidative phosphorylation) and biosynthetic capability (Sutherland et al., 2005). Furthermore, EGCG has been shown to restore post-ischemic Na^+^-K^+^-ATPase activity in the brain and enhance its functional coupling with the astrocytic glutamate transporter-1 (GLT-1), thereby accelerating glutamate clearance from the synaptic cleft (Liu et al., 2025).

EGCG also exerts precise regulatory effects on mitochondrial dynamics equilibrium. It prevents mitochondrial calcium overload by inhibiting voltage-gated calcium channels (VGCCs), thereby regulating key proteins involved in mitochondrial fusion and fission—such as downregulating fission-promoting proteins dynamic-related protein 1 (Drp1)/fission protein 1 (Fis1) and upregulating fusion-promoting proteins optic atrophy 1 (OPA1)/mitofusins. Consequently, this reverses excessive mitochondrial fragmentation following ischemic injury and preserves their normal reticular structure (Chen et al., 2018). Simultaneously, EGCG may regulate autophagic flux to facilitate the clearance of damaged mitochondria, as evidenced by the modulation of Beclin-1 and Atg5 expression (Chen et al., 2018). However, direct evidence for PTEN induced kinase 1 (PINK1)/Parkin-mediated mitophagy in stroke models remains limited.

At the signaling pathway level, EGCG has been shown to modulate ERK signaling. Qin et al. utilized a rat cardiac arrest/cardiopulmonary resuscitation (CA/CPR) model and found that EGCG treatment exhibited effects similar to those of the extracellular signal-regulated kinase (ERK) inhibitor PD98059, specifically reducing p-ERK expression, restoring mitochondrial function, and improving neurological outcomes in animals (Qin et al., 2019).

Furthermore, EC has been investigated primarily in combination with other compounds. Studies have confirmed that the combined application of EC and quercetin can cooperatively attenuate oxygen-glucose deprivation (OGD)-induced neuronal death (Nichols et al., 2015). This improvement is accompanied by increased phosphorylation levels of cAMP response element-binding protein (CREB) and upregulation of its target genes—Bcl-2, which promotes neuronal survival, and peroxisome proliferator-activated receptor gamma coactivator 1-alpha (PGC-1α), which enhances mitochondrial biogenesis. Both compounds converge on NOS and CREB by activating Akt and calcium-mediated signaling pathways, thereby synergistically improving neuronal mitochondrial performance. However, the specific contribution of EC versus quercetin in this synergistic effect remains unclear, and no studies have evaluated EC as a single agent for mitochondrial protection in stroke models.

Other catechins have received limited attention. In OGD-exposed C6 glioma cells, catechin, EC, and EGCG protected against ischemic swelling via mitochondrial mechanisms (Panickar et al., 2009).

In summary, the evidence for catechin-mediated mitochondrial protection is overwhelmingly derived from EGCG studies, with limited data available for EC and catechin. EGCG has been demonstrated to preserve respiratory chain activity, regulate mitochondrial dynamics, prevent calcium overload, and modulate ERK signaling. Catechins preserve BBB integrity by inhibiting MMP-9 and tight junction degradation, though age may modulate this effect. They also protect mitochondria by maintaining electron transport chain activity, regulating fusion/fission balance, preventing calcium overload, and promoting mitophagy. These dual actions stabilize the neurovascular unit and energy metabolism (Figure 4). While catechins are known to protect the BBB, the extent of this benefit varies across different disease models and age groups in animals. It remains uncertain whether barrier stabilization serves as a primary neuroprotective mechanism or a secondary effect with limited clinical significance. Moreover, few studies have utilized aged animals with comorbidities such as hypertension, diabetes, or hyperlipidemia. These conditions significantly disrupt BBB integrity and may attenuate the therapeutic effects of catechins, highlighting a notable limitation in current research.

**FIGURE 4:**
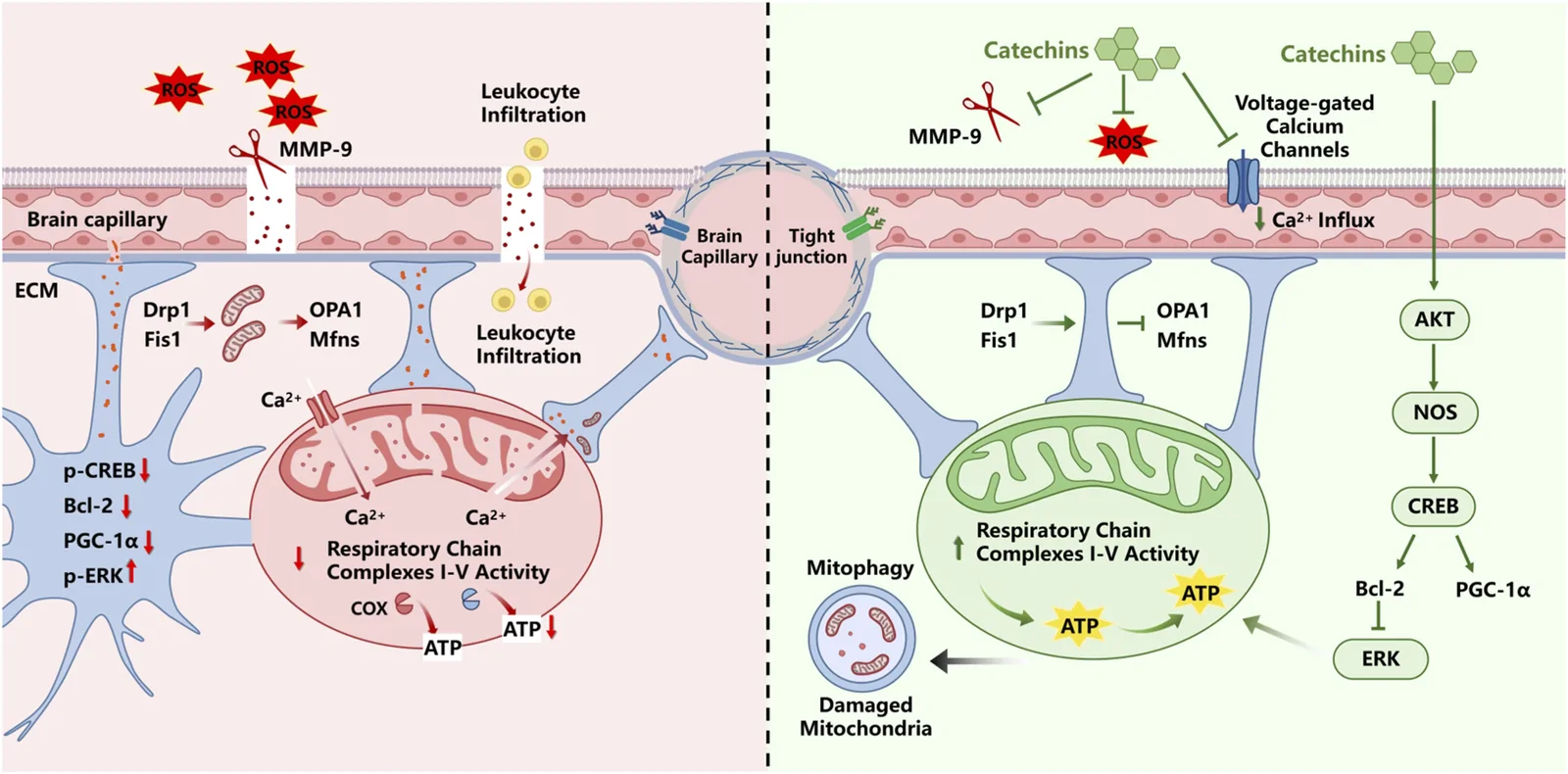
Catechins play a crucial role in preserving the integrity of the BBB and maintaining mitochondrial function. They protect the BBB by inhibiting the degradation of tight junction proteins, while also reducing the activity of MMP-9. Additionally, catechins modulate neuroinflammation by influencing the eNOS and iNOS pathways, thereby reducing oxidative stress and suppressing pro-inflammatory signaling cascades to maintain BBB function.

### 4.4 Promoting repair and regeneration: angiogenesis and neurogenesis

Beyond their acute neuroprotective effects, catechins actively contribute to long-term brain repair through mechanisms such as angiogenesis, neurogenesis, and synaptic plasticity. Park et al. employed proteomic techniques to demonstrate that rats subjected to MCAO and treated with EGCG exhibited significant alterations in the expression of various proteins associated with energy metabolism and neuronal protection, including isocitrate dehydrogenase, dynamin-related protein 1, and γ-enolase. These findings indicate that EGCG plays a role in post-ischemic neuroprotective mechanisms by modulating endogenous repair-related proteins (Park et al., 2021a).

#### 4.4.1 Angiogenesis via Nrf2/vascular endothelial growth factor (VEGF) axis

EGCG has been most extensively studied for its pro-angiogenic effects. EGCG treatment significantly increases neovascular density (Ki67/CD31-positive) in the ischemic penumbra and activates the VEGF-VEGFR2 pathway. This pro-angiogenic effect depends on Nrf2 activation, as blocking Nrf2 completely abolishes the pro-angiogenic and neuroprotective effects of EGCG (Bai et al., 2017). This indicates that EGCG promotes the establishment of collateral circulation and the recovery of blood supply by activating the Nrf2-VEGF axis, providing a crucial material basis for the repair of ischemic tissues.

Furthermore, ECG has also been investigated for its effects on angiogenesis, although to a lesser extent. In human brain microvascular endothelial cells (HBMVECs) subjected to OGD/R, ECG and EGCG regulated VEGF, Bcl-2, Bax, LC3B, caspase-3, mTOR, and Beclin-1, promoting angiogenesis, proliferation, tube formation, and migration while inhibiting apoptosis and autophagy (Fu et al., 2019).

In summary, the evidence for catechin-mediated angiogenesis is primarily derived from EGCG studies, with limited data available for ECG. The Nrf2/VEGF axis has been established as a key mechanism for EGCG, but whether other catechins act through similar pathways requires further investigation.

#### 4.4.2 Neurogenesis and neuronal differentiation

EGCG is the most extensively studied catechin for its effects on neurogenesis and neuronal differentiation. Even when delayed treatment is initiated 14 days after cerebral ischemia, EGCG persistently promotes proliferation and migration of neural progenitor cells in the subventricular zone and their differentiation into neurons. This process relies on Akt activation and a pro-regenerative microenvironment formed by EGCG-induced M2-type microglia (Zhang et al., 2017).

Additionally, EGCG potentiates the action of neurotrophic factors. Extremely low concentrations of the compound bind to the 67LR, triggering H_2_O_2_ production and enhancing brain-derived neurotrophic factor (BDNF) signaling through its receptor tropomyosin receptor kinase B (TrkB), thereby strongly promoting synaptic growth (Gundimeda et al., 2014). Regarding reversing post-injury regenerative inhibition, catechins precisely target key signaling axes. Following SAH, pretreatment with EGCG effectively reverses injury-induced neurite outgrowth inhibition, rescues abnormal neuronal morphology, and ultimately improves neurological function scores by scavenging excessive ROS and suppressing the downstream integrin subunit beta 1 (integrin β1)/focal adhesion kinase (FAK)/p38 signaling axis (Zhang et al., 2021).

Mixed green tea polyphenol extracts have also demonstrated neurogenic effects, although the specific contributions of individual components remain unclear. Studies have confirmed that green tea polyphenol extracts and their primary component EGCG can significantly enhance NGF-induced neurite outgrowth in PC12 cells, while upregulating the expression of neuronal maturity markers neurofilament-L and growth-associated protein-43 (GAP-43) (Gundimeda et al., 2010).

In summary, EGCG promotes neural progenitor cell proliferation and migration, enhances BDNF/TrkB signaling, and reverses injury-induced neurite outgrowth inhibition through the integrin β1/FAK/p38 pathway. While mixed green tea polyphenol extracts also exhibit benefits, the specific contributions of individual components remain unclear.

#### 4.4.3 Synaptic plasticity and glial support

EGCG has been shown to directly improve synaptic plasticity and signal balance at the level of neural network function. Electrophysiological studies demonstrate that appropriate doses of EGCG enhance long-term potentiation in the hippocampal Schaffer collateral-CA1 pathway and restore the balance between excitatory and inhibitory neurotransmitters, such as glutamate and GABA, following cerebral ischemia (Ding et al., 2012).

ECG has been investigated primarily for its effects on astrocytes, a critical component of the neurovascular unit that supports neuronal survival and synaptic function. ECG significantly promotes the maturation of astrocytes and enhances two of their core functions in a time-dependent manner, namely, the capacity to clear excitatory glutamate and the secretion level of neurotrophic factor S100 calcium-binding protein B (S100B) (Abib et al., 2008). This indicates that catechins can indirectly support neuronal survival, synaptic plasticity, and repair processes by optimizing the microenvironment surrounding neurons.

In summary, the evidence for catechin-mediated synaptic plasticity and glial support is derived from EGCG and ECG studies. Catechins promote post-stroke repair by stimulating angiogenesis via Nrf2/VEGF, enhancing neurogenesis and neuronal differentiation through BDNF/TrkB and 67LR signaling, and improving synaptic plasticity. These effects, supported by M2 microglia and reactive astrocytes, contribute to long-term functional recovery (Figure 5).

**FIGURE 5:**
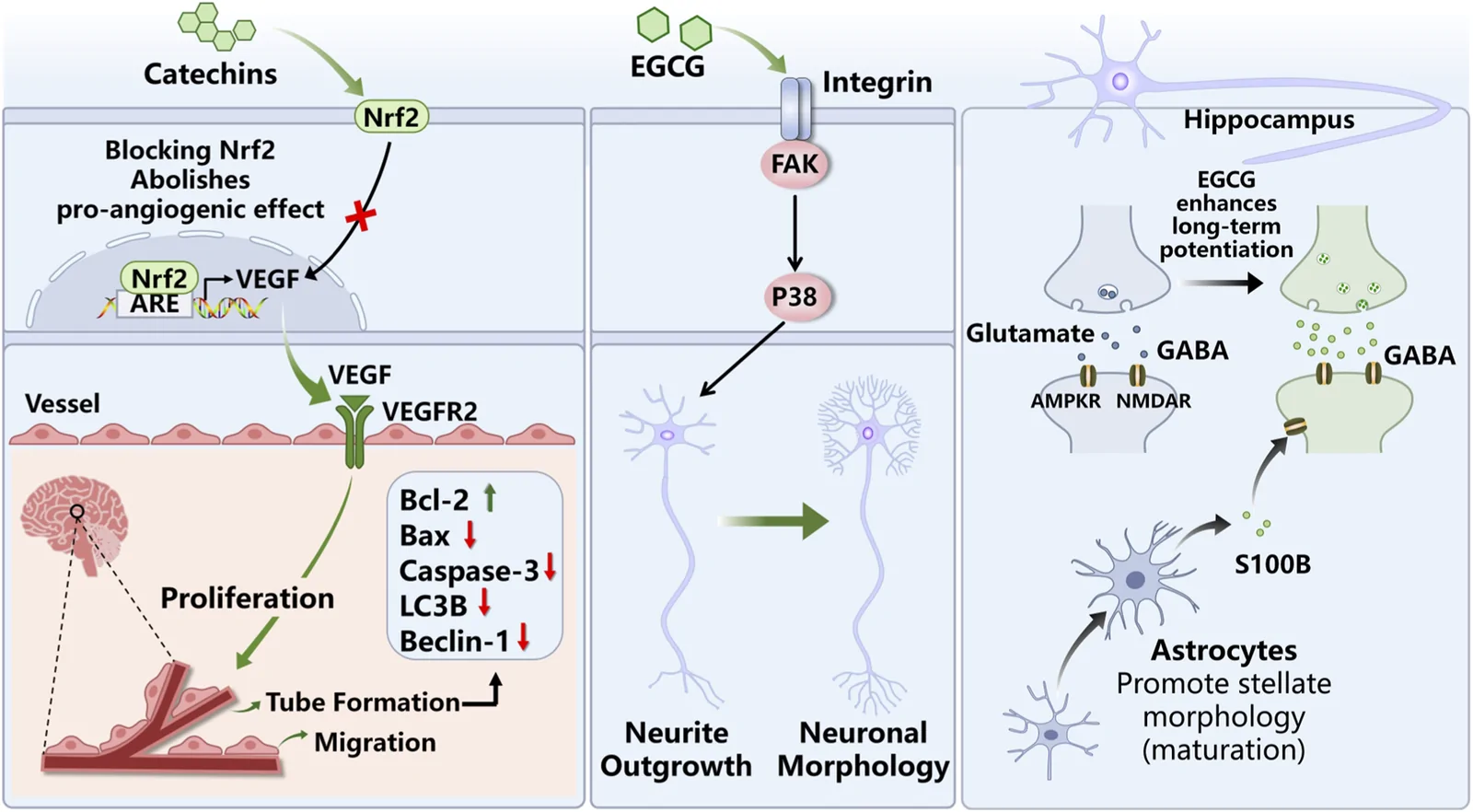
Catechins play a significant role in promoting repair and regeneration following a stroke. They exhibit multifaceted neuroprotective effects, inhibiting the Nrf2/HO-1 pathway to reduce oxidative stress and apoptosis. Concurrently, catechins promote angiogenesis through the VEGF/VEGFR2 axis. Moreover, EGCG activates the Integrin-FAK-p38 pathway, enhancing neurite outgrowth and neuronal morphology. Additionally, catechins modulate neurotransmission and facilitate astrocyte maturation in the hippocampus.

## 5 Evidence from preclinical models (*in vivo* and *in vitro*)

The neuroprotective effects of catechins against stroke have been extensively evaluated using various established *in vitro* and *in vivo* preclinical models. These models replicate key pathophysiological features of ischemic and hemorrhagic stroke, providing a platform to assess the therapeutic potential of compounds such as EGCG, EC, and catechin hydrate. Given the multifactorial nature of stroke pathophysiology and the challenges of translating preclinical findings into clinical treatments, it is critical to carefully select appropriate models—each with distinct advantages and limitations—for evaluating the multi-target actions of catechins (Kumar et al., 2016; Thaysen et al., 2024). Detailed experimental parameters such as concentrations, exposure times, and specific treatment regimens are summarized in Supplementary Table S1.

### 5.1 *In Vitro* models

A range of cell lines and primary cultures have been employed to model stroke-related injury *in vitro*, focusing on oxidative stress, excitotoxicity, neuroinflammation, and OGD. Each model system offers distinct trade-offs between experimental convenience and biological relevance.

#### 5.1.1 Neuronal cell models

PC12 cells (derived from rat pheochromocytoma) are widely used to model neuronal responses to ischemic and oxidative stress. Injury is induced by exposure to H_2_O_2_, glutamate, cobalt chloride (CoCl_2_, mimicking hypoxia), SNP (an NO donor), or OGD/R. PC12 cells are valued for their rapid proliferation, ease of culture, and ability to differentiate into neuron-like cells upon NGF stimulation, making them highly amenable to high-throughput screening of neuroprotective agent**s** (Chua and Lim, 2021). However, as an immortalized cell line derived from a tumor, PC12 cells exhibit significant differences from primary neurons; for instance, they lack functional NMDA receptors, which limits their utility in studying excitotoxicity mechanisms via NMDA-mediated pathways (Edwards et al., 2007). Additionally, repeated passaging may introduce genetic drift, and results obtained with PC12 cells require validation in primary neuronal cultures (Kinarivala et al., 2017; Chua and Lim, 2021).

HT22 cells, which are derived from mouse hippocampal neurons, are commonly subjected to glutamate or OGD/R to induce excitotoxic and ischemic injury. As an immortalized cell line, HT22 cells provide reproducibility and ease of culture; however, similar to PC12 cells, they do not fully replicate the neuronal heterogeneity and synaptic connectivity observed in primary neurons (Klenke et al., 2020; Murphy et al., 2025).

Primary rat hippocampal and cortical neurons, obtained directly from animal tissue, maintain their native morphology, receptor expression, and functional synaptic networks, thus more closely reflecting the *in vivo* neuronal environment (Harrill et al., 2015). These primary neurons are employed in OGD/R or excitotoxicity models using NMDA or glutamate to assess direct neuronal injury. Nevertheless, primary neuronal cultures are labor-intensive, necessitate specialized culture conditions and fresh isolation from animal tissue, possess limited proliferative capacity, and exhibit batch-to-batch variability (Aggarwal et al., 2025).

SH-SY5Y cells, derived from human neuroblastoma, provide the advantage of a human genetic background, rendering them more translationally relevant compared to rodent-derived cell lines. They are frequently differentiated into neuron-like phenotypes and subjected to OGD/R to model ischemic injury (Jiang et al., 2024).

#### 5.1.2 Glial and neurovascular unit models

Primary rat astrocytes and mouse primary astrocytes (wild-type and Nrf2 knockout) are crucial for investigating the contributions of glial cells to stroke pathology. Isolating these cells from genetically modified animals, such as Nrf2 knockout mice, facilitates the dissection of specific molecular pathways. Additionally, exposure to hemoglobin is employed to model astrocyte toxicity induced by ICH (Lan et al., 2017). However, the isolation of astrocytes is a time-consuming process, and these cells can be passaged only a limited number of times.

BV-2 microglial cells (immortalized mouse microglia) retain most of the morphological and functional characteristics of primary microglia. They express key microglial markers (F4/80, CD11b, Iba1) and respond to inflammatory stimuli such as LPS and hypoxia. When treated with LPS, CoCl_2_, or hemoglobin, these cells are widely utilized to model neuroinflammatory responses, providing an unlimited and cost-effective supply that reduces the necessity for continuous primary cell preparations and animal experimentation (Henn et al., 2009). However, as an immortalized cell line, BV-2 cells do not fully replicate the complexity of primary microglia *in vivo*. Furthermore, their murine origin may limit direct applicability to human diseases, and the gene upregulation in response to LPS is less pronounced compared to that observed in primary microglia (Branca et al., 2015; He et al., 2018).

C6 glioma cells (rat glial cell line) are an immortalized astrocytic cell line that serves as a valuable model for investigating glial swelling and ischemic injury under conditions of OGD or hemoglobin treatment (Jung et al., 2010; Panickar and Anderson, 2011). These C6 cells are relatively easy to culture and yield consistent results (Zhang and Zhang, 2015). However, being a tumor-derived cell line, they may display altered physiological responses when compared to primary astrocytes (Ringel and Plesnila, 2008).

HBMVECs are utilized in OGD/R models to evaluate BBB integrity, angiogenesis, and endothelial cell survival (Yi et al., 2020; Wang et al., 2022; Yang et al., 2023). Originating from human tissue (Lippmann et al., 2020), HBMVECs enhance the translational relevance of research findings. Nevertheless, they present greater challenges in culture maintenance and have a limited number of passages available (Lippmann et al., 2020; Monteiro et al., 2024).

#### 5.1.3 Other models

The hyperbaric oxygen chamber model, specifically the *in vitro* red blood cell model, is employed to evaluate the effects of catechins on free radical-induced hemorheological abnormalities. This model provides a straightforward system for investigating oxidative damage to blood components; however, it does not accurately replicate neuronal injury responses (Chen et al., 2011).

### 5.2 *In Vivo* animal models

Rodent models, primarily consisting of rats and mice, are the most commonly utilized *in vivo* systems for simulating various types of strokes and assessing the therapeutic efficacy of catechins. The use of rodents presents several advantages. They are relatively cost-effective, easy to handle, possess well-characterized genetics, and their cerebral vasculature closely resembles that of humans in critical aspects. Nevertheless, no single animal model can fully capture the complexity and heterogeneity of human stroke, which underscores the necessity of employing multiple models during preclinical evaluations (Fluri et al., 2015).

#### 5.2.1 Ischemic stroke models

MCAO/R: This model is the most widely utilized animal model for focal cerebral ischemia. It involves either transient (tMCAO) or permanent (pMCAO) occlusion, typically achieved using an intraluminal filament or thromboembolic approach, often followed by reperfusion (Fluri et al., 2015; Zeng et al., 2023). The MCAO model is employed in various species, including Sprague-Dawley rats, wistar rats, C57BL/6 mice, and gerbils. This model closely simulates human ischemic stroke (Fluri et al., 2015), characterized by reliable and reproducible infarcts that predominantly affect the cortex and striatum (Morris et al., 2016). It allows for controlled occlusion duration (transient vs. permanent) and facilitates the assessment of reperfusion injury—a clinically significant phenomenon following thrombolysis or thrombectomy (Fluri et al., 2015; Sutherland et al., 2016). However, the intraluminal filament technique necessitates surgical expertise, and variations in filament size, insertion depth, and occlusion time can significantly influence infarct size and reproducibility (Morris et al., 2016). Most studies are conducted using young, healthy animals, while human stroke typically affects elderly patients with comorbidities such as hypertension and diabetes, which diminishes the translational potential of these findings (Fluri et al., 2015; McCann and Lawrence, 2020). Additionally, the therapeutic window for neuroprotective agents in animal models can extend up to 12 h, often exceeding the approximately 3-h window observed in humans, potentially leading to an overestimation of drug efficacy (Fluri et al., 2015).

##### 5.2.1.1 Global cerebral ischemia models

Bilateral common carotid artery occlusion (2-VO): This model induces transient global ischemia, primarily in gerbils or rats. The anatomical structure of the circle of Willis in gerbils is incomplete, and bilateral occlusion of the common carotid arteries can lead to significant cerebral ischemic injury, making gerbils an ideal species for this model (León-Moreno et al., 2020). This approach mimics cardiac arrest or severe hypotension but does not replicate focal ischemic stroke, which is the most common form of stroke in humans (Smith et al., 1984; Krafft et al., 2012).

CA/CPR model: Utilized in rats, this model simulates global cerebral I/R injury following cardiac arrest (Katz et al., 1995). It is clinically relevant for studying whole-brain ischemia; however, the injury mechanisms differ from those observed in focal ischemia (Hossmann, 2012). Notably, ERK signaling responses in CA/CPR can oppose those in MCAO (Wang et al., 2004), which may lead to divergent catechin effects across models. Furthermore, despite the CA/CPR model yielding numerous positive experimental outcomes in preclinical studies, many neuroprotective drugs have failed to show clinical benefits in human cardiac arrest patients, highlighting translational challenges (Motta et al., 2025).

##### 5.2.1.2 Hypoxia-ischemia (HI) model

The modified Levine model, which simulates neonatal HI in rats, involves unilateral carotid artery ligation followed by systemic hypoxia. This model effectively mimics neonatal hypoxic-ischemic encephalopathy; however, it is not directly applicable to adult ischemic stroke (Vannucci and Vannucci, 2005; Mallard and Vexler, 2015).

#### 5.2.2 Hemorrhagic stroke models

##### 5.2.2.1 ICH models

There are two primary approaches to induce ICH. One is injection of collagenase, which enzymatically disrupts the basal lamina of blood vessels, leading to diffuse bleeding. The other is injection of autologous blood, which mimics vessel rupture within the brain parenchyma (MacLellan et al., 2008; Wang, 2010; Thangameeran et al., 2023). Both models effectively recreate the essential characteristics of human ICH, demonstrating good face validity (MacLellan et al., 2008; Thangameeran et al., 2023). The collagenase model produces larger and more reproducible hematomas, while the blood injection model more accurately simulates the mass effect of a clotted hematoma (MacLellan et al., 2008). These models facilitate the investigation of secondary brain injury mechanisms, including edema, inflammation, and cell death (Xi et al., 2006; MacLellan et al., 2008). Despite numerous preclinical successes, no treatments tested in phase III clinical trials have shown benefit for ICH patients, raising concerns regarding the predictive value of current ICH models (MacLellan et al., 2008; Kirkman et al., 2011; Withers et al., 2020). The collagenase model may induce greater BBB breakdown and tissue damage compared to clinical ICH, while the blood injection model often underestimates inflammatory responses (MacLellan et al., 2008; Jia et al., 2021). Furthermore, the two models exhibit distinct bleeding profiles and cell death trajectories, suggesting that preclinical assessments should ideally incorporate both models (MacLellan et al., 2008; Kirkman et al., 2011). Neither model fully replicates the severity of clinical ICH, and most studies utilize young, healthy rodents rather than aged or hypertensive animals, which would better represent the patient population (Thangameeran et al., 2023; Wilkinson et al., 2024).

##### 5.2.2.2 SAH model

SAH is typically induced by injecting oxyhemoglobin (OxyHb) or via endovascular filament perforation in mice. EGCG, including its nanoparticle formulation (PEG-PLGA-EGCG), has been tested in this model (Huang et al., 2024). The endovascular perforation model replicates several critical pathophysiological features of clinical SAH, such as an acute increase in intracranial pressure, early brain injury, and delayed cerebral ischemia (Lee et al., 2009). This model is regarded as the most clinically relevant simulation of SAH (Du et al., 2016). However, it necessitates a demanding surgical technique and is associated with high mortality rates, as well as uncontrollable bleeding volumes and other intracranial complications (Alpdogan et al., 2024). Blood injection models provide greater reproducibility but do not exhibit the acute pressure surge characteristic of clinical aneurysm rupture (Lee et al., 2009). Similar to other stroke models, the majority of SAH studies are conducted in young animals without aneurysmal pathology, which limits their translational relevance (Leclerc et al., 2018; Oka et al., 2020).

#### 5.2.3 Other *in Vivo* models

Stroke-prone spontaneously hypertensive rats (SHRSP): This genetic model, derived from Wistar-Kyoto rats through selective breeding, exhibits spontaneous development of severe hypertension. When subjected to salt loading, SHRSP demonstrate a greater than 95% incidence of stroke, predominantly of the hemorrhagic type (Yamagata, 2012). This unique strain has been utilized to evaluate the long-term preventive effects of dietary catechins, such as EGCG (Uchida et al., 1995). SHRSP serves as a clinically relevant model for hypertension-associated stroke, a condition that affects a significant proportion of stroke patients, thereby providing a framework to assess chronic preventive interventions (Nabika et al., 2012). However, the genetic mechanisms underlying hypertension and stroke in SHRSP are complex and not fully understood, suggesting that findings from this model may not be directly extrapolated to non-hypertensive stroke populations (Bailey et al., 2011; Krafft et al., 2012; Nabika et al., 2012).

Permanent focal ischemia in aged mice: Used to assess the efficacy of catechins such as EC in the aging brain (Leonardo et al., 2015). The aging brain exhibits distinct pathophysiological responses to ischemia, which include altered inflammatory and antioxidant signaling that may influence the protective efficacy of various therapeutic agents (Rosenzweig and Carmichael, 2013; Feng et al., 2023). This model offers a more clinically relevant context for investigating stroke therapies in the elderly, who represent the majority of stroke patients (Popa-Wagner et al., 2020; Candelario-Jalil and Paul, 2021). However, the use of aged mice presents challenges, as they are more costly and variable, and breeding or maintaining sufficient numbers for statistical power can be difficult (Miller and Nadon, 2000; Jackson et al., 2017).

### 5.3 Model selection influences catechin efficacy conclusions

The type of stroke model significantly influences the observed neuroprotective effects of catechins, and inconsistent or even conflicting results in the literature may be partially attributed to differences in model species, age, and modeling methods.

One notable example is the divergent role of ERK signaling. In the MCAO model of focal ischemia, p-ERK levels are decreased, and restoration of p-ERK expression confers neuroprotection (Wang et al., 2013; Zhu et al., 2013; Yang et al., 2016). In contrast, in the global ischemia model, excessive ERK phosphorylation contributes to injury, and inhibiting p-ERK improves outcomes (Nguyen Thi et al., 2016; Zheng et al., 2019). EGCG has been shown to reduce p-ERK in the CA/CPR model (Qin et al., 2019), which is protective in that context. However, if the same mechanism were operative in MCAO, it might be detrimental, underscoring the importance of model-specific interpretation.

Similarly, BBB protection by catechins appears to be age-dependent. In young mice, EC preserves BBB integrity following ischemia, whereas in aged mice (12 months), this protective effect is lost, despite preserved neuroprotection through other mechanisms (Leonardo et al., 2013; Leonardo et al., 2015). This finding suggests that conclusions about catechin mechanisms drawn from young animals may not automatically apply to aged populations.

For hemorrhagic stroke, the two ICH models (collagenase vs. blood injection) produce different inflammatory and cell death profiles. Catechin’s Nrf2-dependent protection has been demonstrated in both models (Chang et al., 2014; Lan et al., 2017), but comparative studies are limited. Ideally, preclinical evaluation of catechins in ICH should include both models to capture the full spectrum of secondary injury (MacLellan et al., 2008).

Collectively, the most consistent and model-independent effects of catechins are the activation of Nrf2 signaling and anti-neuroinflammation, whereas BBB protection and ERK modulation are model-dependent and affected by age, ischemia type, and hemorrhagic model. Therefore, the neuroprotective efficacy of catechins must be interpreted in combination with specific experimental models, and future studies should incorporate aged animals, comorbid models, and both sexes to improve clinical translational value.

## 6 Potential clinical significance of catechins

Among various TCM compound formulations, catechin has been widely detected and is recognized as one of the key active components responsible for the cardiovascular and cerebrovascular protective effects of TCM (Zhang et al., 2024). The combination of Chuanxiong (Ligusticum chuanxiong) and Chishao (Paeoniae Radix Rubra) demonstrates significant therapeutic efficacy in treating focal cerebral ischemia. Eight major components were identified in their extracts, with catechin present at relatively high levels. Studies have shown that the Chuanxiong-Chishao combination can significantly enhance the activities of SOD, catalase, and glutathione peroxidase (GSH-Px) in the serum of MCAO model rats, while markedly reducing the levels of MDA and lipid peroxides (LPO). In addition, the protein expression levels of endoplasmic reticulum stress-related factors PKR-like endoplasmic reticulum kinase (PERK), X box-binding protein-1 (XBP-1), activating transcription factor-6 (ATF-6), and CHOP were significantly decreased, while the expressions of GRP78 were upregulated (Gu et al., 2016).

Catechins have also demonstrated favorable synergistic effects when combined with other medications in preclinical studies. For instance, EGCG can extend the narrow therapeutic time window of recombinant tissue plasminogen activator (rt-PA) in rat models of MCAO, with combined administration significantly improving neurobehavioral deficits even when given 4 h after ischemia onset (You, 2016). However, clinical evidence for catechins in stroke remains extremely limited. To date, only one randomized controlled trial has evaluated EGCG in stroke patients. In that study, 371 patients with acute ischemic stroke were randomly assigned to receive either rt-PA combined with EGCG or rt-PA combined with placebo. EGCG was administered as a 500 mg intravenous infusion on day 1, followed by 500 mg daily for 7 days. The primary endpoint was the National Institutes of Health Stroke Scale (NIHSS) score at day 1 and day 7; secondary endpoints included plasma levels of MMP-2 and MMP-9. In patients with an onset-to-treatment time (OTT) of 3–5 h, EGCG significantly improved NIHSS scores and reduced MMP-2 and MMP-9 levels compared with placebo, with no serious adverse events reported. These findings suggest that EGCG may extend the therapeutic window of rt-PA in patients with delayed OTT. However, the trial was conducted at a single center with a relatively short follow-up period (7 days), and the sample size for subgroup analyses was limited. Larger, multicenter trials with longer follow-up are needed to confirm these preliminary findings (Wang and You, 2017).

To further enhance the bioavailability of EGCG and its delivery efficiency to brain tissues, researchers have developed various novel formulations. PEGylated PLGA EGCG nanoparticles (EGCG-NPs) prepared by the double emulsion method were administered as a pretreatment at a dose of 75 mg/kg/d for 7 days before SAH, combined with nimodipine at a dose of 10 mg/kg/d administered 30 min after SAH. This approach demonstrated significant synergistic anti-neuronal death effects by inhibiting oxidative stress, calcium overload, mitochondrial dysfunction, and autophagy (Yang et al., 2022). Additionally, EGCG solid lipid nanoparticles developed using microemulsion technology significantly improved the delivery efficiency of EGCG to brain tissues (Kaur et al., 2019). Additionally, EGCG-stabilized selenium nanoparticles functionalized with the OX26 antibody and the NMDA antagonist MK-801 have been shown to reduce infarct volume, lower glutamate and calcium levels, and improve cognitive outcomes after cerebral ischemia (Akcay et al., 2025). Catechin-based polyphenol nanoparticles surface-modified with thiol-terminated polyethylene glycol (CNPs@PEG) effectively maintain BBB integrity, alleviate cerebral edema, significantly improve survival rates in mice with ICH, and ameliorate neurological deficits by chelating iron ions, enhancing tissue antioxidant capacity, reducing oxidative stress, and inhibiting iron deposition (Zeng et al., 2025). Another study employed the hydration-film extrusion method using phosphatidylcholine or phosphatidylserine as membrane materials to prepare EGCG liposomes with or without vitamin E. In the LPS-induced BV-2 microglial cell model, EGCG liposome intervention decreased the production levels of NO and tumor TNF-α in LPS-stimulated BV-2 cells, exerting an anti-inflammatory protective effect (Cheng et al., 2021).

## 7 Discussion

This review examines the neuroprotective role of catechins, particularly EGCG, in the context of stroke. It reveals that dosage, cell-type specificity, and variations in experimental models are three critical variables that must be considered when interpreting their effects. An in-depth exploration of these factors enhances our understanding of the multifaceted nature of catechin actions and offers vital references for the design of future research and clinical applications.

### 7.1 Critical appraisal and controversies

Consistent evidence identifies Nrf2 activation as a key protective pathway triggered by catechin treatment. EGCG produces distinct biological outcomes at varying concentrations, in accordance with the principles of hormesis. At low concentrations, EGCG effectively clears ROS, thereby reducing oxidative damage. However, elevated levels of EGCG increase the abundance of HO-1 transcripts. These alterations may reflect cellular stress signals rather than protective regulatory cascades. Furthermore, sustained Nrf2 activation presents potential risks, including a diminished antioxidant capacity and an increased risk of tumor development. Few studies related to stroke have thoroughly examined these adverse outcomes.

Numerous published studies emphasize the benefits of catechin-mediated NF-κB inhibition. However, researchers should exercise caution when drawing overly simplistic conclusions. NF-κB serves two distinct functions within nervous tissue. Brief NF-κB activity promotes the production of neurotrophic molecules essential for tissue repair, whereas prolonged NF-κB signaling exacerbates secondary neural damage. Current research does not comprehensively elucidate how catechins modulate NF-κB activity over time, as most experimental observations focus solely on acute disease stages. Consequently, the long-term effects of this regulatory shift on post-stroke tissue reconstruction remain largely unexplored.

### 7.2 Pretreatment versus post-stroke treatment

A critical issue in interpreting the preclinical evidence for catechins is the distinction between pretreatment (preconditioning) and post-stroke therapeutic administration. As summarized in Supplementary Table S1, a substantial proportion of studies administered the intervention before stroke induction. These include investigations involving catechin hydrate (Ashafaq et al., 2012), EGCG in SAH models (Chen et al., 2017; Huang et al., 2024), and various *in vitro* preconditioning experiments (Sutherland et al., 2005; Fu and Koo, 2006; Gundimeda et al., 2012; Chen et al., 2016). For instance, Ashafaq et al. administered catechin hydrate for 21 days before the induction of MCAO (Ashafaq et al., 2012), Huang et al. administered EGCG-NPs for 7 days before SAH (Huang et al., 2024), and Chen et al. administered EGCG for 14 days prior to SAH (Chen et al., 2017). While these studies provide valuable insights into preconditioning mechanisms, they do not accurately model the clinical scenario in which treatment is initiated after stroke onset. This distinction is critical because preconditioning activates endogenous protective pathways before injury, whereas post-stroke treatment must counteract ongoing pathological cascades within a narrow therapeutic window. Moreover, agents that show efficacy in preconditioning models often fail when administered after injury (Endres et al., 2008; Fluri et al., 2015).

Among the studies reviewed, only a limited number have evaluated catechins in clinically relevant post-stroke treatment regimens, including delayed EGCG administration for neurogenesis (Zhang et al., 2017), immediate post-MCAO injection for anti-apoptosis (Nan et al., 2018; Park et al., 2020), EGCG combined with rt-PA 4 h after MCAO (You, 2016; Wang and You, 2017), and post-resuscitation EGCG in a CA/CPR model (Qin et al., 2019). Collectively, the evidence for post-stroke therapeutic administration is substantially weaker than that for preconditioning. Future studies should prioritize clinically relevant post-stroke treatment regimens with appropriate dose optimization and time-window assessment. Positive results from preconditioning studies should not be overinterpreted as evidence of therapeutic efficacy.

### 7.3 Precise dose–response relationship and therapeutic window

Current evidence clearly demonstrates the dose-dependent characteristics of EGCG’s effects. *In vivo* ischemia models, EGCG exhibits a distinct dose-response relationship. Lee et al. found that in a gerbil global cerebral ischemia model, EGCG dose-dependently reduced hippocampal CA1 neuronal death, with the most significant effect observed at a dose of 50 mg/kg (Lee et al., 2000). Multiple studies using the MCAO model have also confirmed that intraperitoneal injection of 50 mg/kg EGCG effectively reduces infarct volume and improves neurological deficits (Park et al., 2020; 2024). Collectively, these studies demonstrate that at the whole-animal level, a specific dose of EGCG (e.g., 50 mg/kg) can effectively mitigate ischemic brain injury, with preliminary evidence confirming its safety—research indicates that a dose of 90 mg/kg shows no significant toxicity in adult rats (Kao et al., 2000).

However, the neuroprotective effects of EGCG do not exhibit unlimited enhancement with increasing doses. *In vitro* studies provide a crucial caveat. At concentrations exceeding 200 µM (approximately 100 μg/mL), EGCG demonstrates cytotoxic effects on normal human epithelial cells (Weisburg et al., 2004). This toxicity is attributed to its pro-oxidant activity at high concentrations, whereby EGCG transitions from an antioxidant to a pro-oxidant, inducing ROS generation and subsequent cellular damage. This phenomenon is also observed in tumor cell studies. EGCG can selectively kill tumor cells by inducing oxidative stress while having minimal impact on normal cells (Min et al., 2012; Tao et al., 2015). Thus, the dosage window of EGCG acts like a double-edged sword. Too low a dose may be ineffective, whereas too high a dose could shift from neuroprotection to neurotoxicity. This presents a core challenge for clinical application, namely, how to precisely determine the optimal therapeutic window that maximizes efficacy while avoiding toxicity.

The contradictory reports in the literature regarding the effects of EGCG, particularly its dual role in apoptosis regulation (both pro-apoptotic and anti-apoptotic), are likely attributable to the interplay between dosage factors, cellular states, and model variations. Zheng et al. observed pro-apoptotic effects of EGCG, manifested as inhibition of ATP synthase activity and induction of mPTP opening (Zheng and Ramirez, 2000). In contrast, studies by Dragicevic and Krishnan demonstrated that EGCG could maintain mitochondrial function and restore Bcl-2 protein levels, thereby exerting anti-apoptotic effects (Dragicevic et al., 2011; Krishnan et al., 2014). This seemingly paradoxical outcome was reconciled through a dose-dependent explanation in MCF-7 cell studies. 50 μM EGCG induced apoptosis by increasing the Bax/Bcl-2 ratio and activating JNK and caspase pathways, whereas higher concentrations (100–400 µM) shifted cell death from apoptosis to necrosis due to acute ATP depletion (Hsuuw and Chan, 2007). This suggests that EGCG’s regulatory direction on apoptosis may depend on cellular energy levels and oxidative stress status determined by its concentration gradient.

Cell type specificity is crucial (Chung et al., 2003). EGCG primarily induces apoptosis to exert its anticancer effects in gastrointestinal cancer cell lines, while it demonstrates protective effects against oxidative stress and neurotoxicity in neuron-like PC12 cells (Raza and John, 2005; Li et al., 2006). This discrepancy may arise from differences in endogenous antioxidant enzyme activities (such as catalase) and the signaling pathway backgrounds among various cell types. In oral cancer cells, EGCG induces mitochondrial oxidative stress by inhibiting the SIRT3 signaling pathway; however, it does not exhibit this effect in normal keratinocytes and may even enhance SIRT3 activity (Tao et al., 2015).

### 7.4 The impact of model differences on research conclusions

The selection of ischemia models significantly impacts the interpretation of the effects of EGCG. The focal cerebral ischemia model, such as the MCAO, which replicates approximately 80% of human ischemic stroke cases, is the most frequently employed model for assessing neuroprotective agents (Chiang et al., 2011). Multiple studies have confirmed that EGCG exerts protective effects by modulating the caspase-3, PARP (Park et al., 2020), hippocalcin (Park et al., 2024), and Nrf2 pathways (Bai et al., 2017). However, in global cerebral ischemia models induced by CA/CPR, the injury mechanisms may differ from those in MCAO. Research indicates that brain injury following CA/CPR is associated with excessive phosphorylation of ERK, and inhibition of p-ERK can improve outcomes (Nguyen Thi et al., 2016; Zheng et al., 2019). Conversely, some MCAO studies have reported decreased p-ERK expression, with restoration of its levels proving beneficial instead (Wang et al., 2013; Zhu et al., 2013; Yang et al., 2016). This differential response in signaling pathways suggests that EGCG may target distinct mechanisms or exert divergent effects in different models, which partially explains the variability of conclusions across studies.

### 7.5 Clinical translation implications and future research directions

However, it must be acknowledged that clinical evidence for catechins in stroke remains preliminary, derived from a single small trial of EGCG (Wang and You, 2017) with short follow-up and no data available for other monomers. The following future directions should therefore be interpreted with this limitation in mind. Notwithstanding the limited clinical evidence, the mechanistic and preclinical analyses presented above indicate that catechins, particularly EGCG, exhibit significant potential in the treatment of stroke. However, several issues must be addressed to facilitate clinical translation. First, determining the precise dosage is paramount. Future research should concentrate on establishing the safe and effective dosage range of EGCG for neuroprotection in humans through pharmacokinetic and pharmacodynamic studies conducted under clinically relevant complex pathological conditions, such as concurrent hyperglycemia and hypertension. Additionally, it is essential to explore the correlation between blood concentration and efficacy/toxicity. Second, improving formulations is crucial to overcoming current bottlenecks. Given the dual challenges of high-dose EGCG’s toxicity and its inherently low bioavailability, the development of novel delivery systems, such as solid lipid nanoparticles, PEGylated PLGA nanoparticles, and liposomes, has proven to be an effective strategy. These formulations not only enhance EGCG’s bioavailability and brain targeting but also facilitate sustained release, thereby promising to reduce effective doses while improving efficacy and minimizing toxicity. Finally, combination therapy strategies demonstrate considerable potential. The co-administration of EGCG with rt-PA can extend the therapeutic time window, while its combination with nimodipine produces synergistic effects against neuronal death in SAH models. Collectively, these preclinical observations suggest that utilizing EGCG as an adjunct to existing therapies, or implementing personalized treatment for specific patient populations, such as those with particular stroke subtypes or genetic backgrounds, within the framework of precision medicine may represent a more feasible pathway for clinical translation.

## 8 Conclusion

Catechins demonstrate multi-target neuroprotective effects against stroke by suppressing oxidative inflammation, inhibiting cell death, preserving the BBB and mitochondria, and promoting repair mechanisms. Notably, the mechanistic and preclinical evidence base for these effects is predominantly derived from EGCG studies, with comparatively limited data available for other monomers such as EC, ECG, and catechin. The extent to which findings from EGCG can be generalized to the entire catechin family remains to be established, and future investigations should prioritize comparative studies of individual monomers. The efficacy of these compounds is dependent on dosage, age, and the specific model used, with Nrf2 activation identified as the most significant mechanism. From a translational perspective, overcoming low oral bioavailability via nanoparticle-based delivery strategies and verifying therapeutic efficacy in aged as well as comorbid animal models constitute key research priorities. Of equal importance is the critical distinction between pre-conditioning pretreatment and post-stroke therapeutic administration. To better mirror real-world clinical scenarios, future experimental designs should give priority to post-stroke treatment regimens when interpreting existing preclinical data.

## Glossary

**67LR**: 67-kDa laminin receptor
**AGE**: Advanced glycation end product
**AMPK**: α-amino-3-hydroxy-5-methyl-4-isoxazolepropionic acid
**AP-1**: Activator protein-1
**APP**: Amyloid Precursor Protein
ARE: Antioxidant Response Element
**ASK1**: Apoptosis Signal-regulating Kinase 1
**ATF**-6: Activating transcription factor-6
**Atg5**: Autophagy Related 5
**Aβ**: Amyloid beta
**BAX**: Bcl-2 associated X protein
**BBB**: Blood-brain barrie
**Bcl**-2: B-cell lymphoma 2
**BDNF**: Brain-derived neurotrophic factor
**CA/CPR**: Cardiac arrest/cardiopulmonary resuscitation
**CD206**: Cluster of differentiation 206
**CG**: (+)-catechin gallate
**CHOP**: C/EBP-homologous protein
**COX-2**: Cyclooxygenase-2
**CREB**: cAMP response element-binding protein
**Drp1**: dynamic-related protein 1
**EC**: (−)-epicatechin
**ECG**: (−)-epicatechin gallate
**EGC**: (−)-epigallocatechin
**EGCG**: (−)-epigallocatechin-3-gallate
**EGCG-NPs**: EGCG nanoparticles
**ERK**: Extracellular signal-regulated kinase
**FAK**: Focal Adhesion Kinase
**Fis1**: Fission protein 1
**GAP-43**: Growth-associated protein-43
**GC**: (+)-gallocatechin
**GCG**: (+)-gallocatechin gallate
**GFAP**: Glial fibrillary acidic protein
**GLT-1**: Glutamate transporter-1
**GRP78**: Glucose-regulated protein 78
**GSH-Px**: Glutathione peroxidase
**GSK-3**: Glycogen synthase kinase-3
**HBMVECs**: Human brain microvascular endothelial cells
**HI**: Hypoxia--ischemia
**HIF-1α**: Hypoxia-inducible factor 1-alpha
**HO-1**: Heme oxygenase 1
**I/R**: Ischemia/reperfusion
**ICH**: Intracerebral hemorrhage
**IL-1β**: Interleukin-1 beta
**IL-6**: Interleukin-6
**iNOS**: Inducible nitric oxide synthase
**integrin β1**: Integrin Subunit Beta 1
**JNK**: Jun-kinase
**Keap1**: Kelch-like ECH-associated protein 1
**LC3B**: Microtubule-associated protein 1 light chain 3 beta
**LPO**: lipid peroxides
**LPS**: lipopolysaccharide
**MAPK**: Mitogen-activated protein kinase
**MCAO**: Middle cerebral artery occlusion
**MCAO/R**: Middle cerebral artery occlusion/reperfusion
**MDA**: Malondialdehyde
**MGO**: Methylglyoxal
**MMP-2**: Matrix metalloproteinase-2
**MMP-9**: Matrix metalloproteinase-9
**mPTP**: mitochondrial permeability transition pore
**mTOR**: Mechanistic Target Of Rapamycin Kinase
**NF-κB**: Nuclear factor ΚB
**NGF**: Nerve growth factor
**NIHSS**: National Institutes of Health Stroke Scale
**NMDA**: N-methyl-D-aspartate
**Nrf2**: Nuclear factor erythroid 2-related factor 2
**OGD**: Oxygen-glucose deprivation
**OGD/R**: Oxygen-glucose deprivation/reoxygenation
**OPA1**: Optic atrophy 1
**OxyHb**: Oxyhemoglobin
**p65**: Nuclear factor kappa-light-chain-enhancer of activated B cells p65 subunit
**p75NTR**: p75 neurotrophin receptor
**PARP**: Poly (ADP-ribose) Polymerase
**PERK**: PKR-like endoplasmic reticulum kinase
**PGC-1α**: peroxisome proliferator-activated receptor gamma coactivator 1-alpha
**PI3K**: Phosphoinositide 3-kinase
**PINK1**: PTEN induced kinase 1
**PKC**: Protein kinase C
**PRISMA**: Preferred Reporting Items for Systematic Reviews and Meta-Analyses
**PS1**: Presenilin 1
**ROS**: Reactive oxygen species
**rt-PA**: Recombinant tissue plasminogen activator
**S100B**: S100 calcium-binding protein B
**SAH**: Subarachnoid hemorrhage
**SHRSP**: Stroke-prone spontaneously hypertensive rats
**SNP**: Sodium nitroprusside
**SOD**: Superoxide dismutase
**STAT1**: Signal transducer and activator of transcription 1
**TCM**: Traditional Chinese medicine
**TLR4**: Toll-like receptor 4
**TNF-α**: Tumor necrosis factor-α
**Trk**: Tropomyosin kinase receptor
**VDAC**: Voltage-dependent anion channel
**VEGF**: Vascular endothelial growth factor
**VGCCs**: Voltage-gated calcium channels
**XBP-1**: X box-binding protein-1
