# Supplementary material for: Catechins in stroke: multi-target mechanisms, preclinical evidence, and translational challenges
Source: Front Pharmacol. 2026 Sep 16;17:1937660. doi: 10.3389/fphar.2026.1937660 (PMC13624970; doi:10.3389/fphar.2026.1937660)
Supplement: Supplementary file 1 [file Table1.docx]

Table S1 Anti-stroke effects of catechins.

| Mechanism | Compound | Treatment regimen | Dosage | Model | Administration | Treatment duration | Main results | References |
| --- | --- | --- | --- | --- | --- | --- | --- | --- |
| Suppressing the oxidative-inflammatory network | EGCG | Pretreatment | In vivo: 0.1%-0.5% | In vivo: SHRSP | In vivo: Dissolved in distilled water (oral) | In vivo:5–51 weeks of age (continuous in drinking water) | EGCG prevented stroke incidence by scavenging free radicals and inhibiting lipid peroxidation. | ^[1]^ |
|  | EGCG | Pretreatment | In vitro: 5-25 µM | In vitro: PC12 cells treated with H₂O₂ | In vitro: Dissolved in culture medium | In vitro: 24 h (co-incubation with H₂O₂) | EGCG increased cellular thiol status, decreased protein carbonyl content, attenuated cell death, increased mitochondrial membrane potential, and reduced TNF‑α levels. | ^[2]^ |
|  | EGCG | Pretreatment | In vivo: 50 mg/kg In vitro: 10‑200 µM | In vivo: Ischemic rat model In vitro: Hippocampal neurons with SNP | In vivo: i.p. In vitro: Dissolved in culture medium | In vivo: Single i.p. bolus (before ischemia)  In vitro: 24 h (co-incubation with SNP) | EGCG protected against ischemic neuronal damage by decomposing peroxynitrite into NO or hydroxyl radical. | ^[3]^ |
|  | EGCG | Pretreatment | In vivo: 40 mg/kg | In vivo: MCAO/R rat model (2 hours ischemia/24 hours reperfusion ) | In vivo: Intraperitoneal injection (i.p.) | In vivo: 3 consecutive daily i.p. injections (last dose 24 h pre-MCAO) | EGCG reduced infarct size and TUNEL‑positive neurons via Nrf2/ARE pathway activation. | ^[4]^ |
|  | EGCG | Post-insult treatment | In vitro: 1, 10, 100 µM | In vitro: PC12 cells treated with glutamate | In vitro: Dissolved in culture medium | In vitro: 14 h (EGCG added 10 h post-glutamate) | EGCG reduced ROS generation and protected against glutamate‑induced oxidative stress. | ^[5]^ |
|  | EGCG | Mixed | In vivo: 25, 50 mg/kg  In vitro: 5 µM | In vivo: Gerbil unilateral cerebral ischemia model  In vitro: Rat hippocampal neurons treated with NMDA, AMPA, or kainate | In vivo: i.p.  In vitro: Dissolved in culture medium | In vivo: 2 boluses (30 min pre + 0 h post)  In vitro: 24 h co-incubation | EGCG attenuated MDA increase, reduced brain edema and infarct volume. | ^[6]^ |
|  | EGCG | Post-stroke treatment | In vivo: 50 mg/kg | In vivo: MCAO/R rat model (2 hours ischemia/24 hours reperfusion) | In vivo: i.p. | In vivo: Single i.p. bolus at reperfusion | EGCG ameliorated TNF‑α, IL‑1β, IL‑6 levels, inhibited NF‑κB/p65 up‑regulation, and reduced COX‑2 and iNOS expression. | ^[7]^ |
|  | EGCG | Pretreatment | In vivo: 12.5, 25, 50 mg/kg In vitro: 50 µM | In vivo: MCAO/R rat model (1.5 hours ischemia/24 hours reperfusion ) In vitro: Rat cortical neurons treated with OGD/R (2 hours OGD/24 hours reperfusion) | In vivo: i.p. In vitro: Dissolved in culture medium | In vivo: Single i.p. bolus pre-MCAO  In vitro: Pre-OGD | EGCG up‑regulated IL‑10 mRNA and protein, and down‑regulated IL‑1β expression. | ^[8]^ |
|  | EGCG | Pretreatment | In vivo: 2 mg/kg/day In vitro: 10 µM | In vivo: APP/PS1 mice In vitro: Rat primary microglia and BV2 cells treated with LPS+Aβ | In vivo: Oral gavage In vitro: Dissolved in culture medium | In vivo: 4 wk daily p.o. In vitro: 1 h pre-incubation (then 7 h with LPS+Aβ) | EGCG inhibited canonical NLRP3 and non‑canonical caspase‑11 inflammasome activation via TLR4/NF‑κB pathway, reducing microglial inflammation and neurotoxicity. | ^[9]^ |
|  | EGCG | Pretreatment | In vitro: 200 µM | In vitro: BV2 microglial cells treated with CoCl₂ | In vitro: Dissolved in culture medium | In vitro: 1 h pre + 8 h co-incubation | EGCG exerted anti‑inflammatory and antioxidant effects by inhibiting NF‑κB and enhancing Nrf2/HO-1 pathway. | ^[10]^ |
|  | EGCG | Post-stroke treatment | In vivo: 10, 20, 30 mg/kg | In vivo: MCAO rat model | In vivo: Oral gavage | In vivo: 7 d daily p.o. (post-MCAO) | EGCG increased CD206 expression (M2 microglia marker). | ^[11]^ |
|  | EGCG | Post-stroke treatment | In vivo: 1 mg/ml, 2 µl In vitro: 10, 20, 40 µM | In vivo: MCAO/R mouse model (1 hours ischemia/28 days reperfusion ) In vitro: SVZ NPCs treated with LPS | In vivo: Slow left ventricular injection In vitro: Dissolved in culture medium | In vivo: 14 daily left ventricular injections (starting at 14 dpi, total 14 d course) In vitro: 7 days (EGCG replenished every 24 h, added after 2 h LPS) | EGCG promoted functional recovery after stroke by enhancing SVZ NPC proliferation and migration, shifting microglia to M2 phenotype, and activating AKT signaling. | ^[12]^ |
|  | EGCG | Pretreatment | In vitro: 5, 10, 20 µM | In vitro: BV‑2 cells treated with LPS | In vitro: Dissolved in culture medium | In vitro: 1 h pre + 20 h co-incubation | EGCG alleviated LPS‑induced protein S‑nitrosylation via Nrf2‑mediated oxidative stress response. | ^[13]^ |
|  | Epicatechin | Mixed | In vivo: 5, 15, 30 mg/kg In vitro: 100 µM | In vivo: MCAO/R mouse model (1.5 hours ischemia/24 or 72 hours reperfusion ) In vitro: Embryonic cortical neurons treated with H₂O₂ | In vivo: Oral gavage In vitro: Dissolved in medium | In vivo: Single oral gavage (90 min before MCAO); Single oral gavage at 3.5 h post-MCAO;Single oral gavage (90 min before NMDA) In vitro: 24 h (EC pre-added, co-incubated with H₂O₂) | Epicatechin activated Nrf2 and increased HO-1 expression, exerting neuroprotection. | ^[14]^ |
|  | Epicatechin | Post-stroke treatment | In vivo: 5, 15, 30 mg/kg In vitro: 50, 100, 200 µM | In vivo: MCAO/R rat model In vitro: Senescent SH‑SY5Y cells treated with OGD/R | In vivo: Femoral vein injection In vitro: Dissolved in medium | In vivo:Single i.v. bolus at reperfusion In vitro:24h post-OGD/R | Epicatechin improved brain morphology and function, increased p‑Nrf2, HO-1, SOD, GSH, reduced infarct volume, attenuated apoptosis, decreased ROS and MDA, and up‑regulated NQO‑1. | ^[15]^ |
|  | (-)-Epicatechin | Mixed | In vitro: 1, 10, 100 µM | In vitro: Mouse primary astrocytes treated with hemoglobin (ICH model) | In vitro: Dissolved in culture medium | In vitro: 1 h pre-Hb+2 h co-incubation/1 h post-Hb+24 h co-incubation | (-)-Epicatechin protected astrocytes against hemoglobin toxicity by up‑regulating Nrf2 and inhibiting AP‑1 activity. | ^[16]^ |
|  | (-)-Epicatechin | Post-stroke treatment | In vivo: 5, 15, 45 mg/kg | In vivo: ICH mouse model | In vivo: Oral gavage | In vivo: 3 doses q24h starting 3 h post-ICH (72 h course) | (-)-Epicatechin reduced lesion volume, improved neurological deficits, reduced cell death and neuronal degeneration, decreased caspase‑3 activity, reduced oxidative damage, increased phase II enzymes and Nrf2 nuclear aggregation. | ^[17]^ |
|  | Catechin hydrate | Pretreatment | In vivo: 20 mg/kg | In vivo: MCAO/R rat model (2 hours ischemia/22 hours reperfusion ) | In vivo: Oral gavage | In vivo: 21 d daily p.o. (pre-MCAO) | Catechin hydrate reduced infarct size, improved neurological deficits, inhibited neuronal loss, down‑regulated iNOS, GFAP, and NF‑κB. | ^[18]^ |
| Anti-apoptosis and autophagy regulation | EGCG | Post-stroke treatment | In vitro: 0.1‑10 µM | In vitro: PC12 cells | In vitro: Dissolved in culture medium | In vivo: 24 h (EGCG added after 48 h serum starved),3 d daily (EGCG added after 3 d serum starved) | EGCG showed “neurorescue” effect via PKC pathway. | ^[19]^ |
|  | EGCG | Pretreatment | In vivo: 50 mg/kg In vitro: 10, 20, 40 µM | In vivo: MCAO rat model In vitro: HT22 cells treated with glutamate | In vivo: i.p. In vitro: Dissolved in culture medium | In vivo: Single i.p. bolus before MCAO In vitro: 1 h pre + 24 h co-incubation (EGCG total 25 h) | EGCG regulated thioredoxin expression and ASK1/thioredoxin binding, exerting neuroprotection. | ^[20]^ |
|  | EGCG | Pretreatment | In vitro: 200 µM | In vitro: PC12 cells treated with SNP | In vitro: Dissolved in culture medium | In vitro: 30 min pre-incubation, then 12 h co-exposure with SNP | EGCG scavenged ROS and regulated cytochrome c, caspases, VDAC, and Bcl‑2 family to protect against apoptosis. | ^[21]^ |
|  | EGCG | Pretreatment | In vitro: 50, 100, 200 µM | In vitro: PC12 cells treated with CoCl₂ | In vitro: Dissolved in culture medium | In vitro: 1 h pre + 24 h co-incubation (CoCl₂) | EGCG prevented cell viability loss, DNA fragmentation, sub‑G1 arrest, mitochondrial membrane potential collapse, cytochrome c release, caspase cascade activation, and normalized Bax/Bcl‑2 ratio. | ^[22]^ |
|  | EGCG | Pretreatment | In vivo: 50 mg/kg | In vivo: MCAO rat model | In vivo: i.p. | In vivo: Single i.p. bolus pre-MCAO | EGCG reduced infarct and apoptosis, decreased caspase‑3 and PARP expression. | ^[23]^ |
|  | EGCG | Post-stroke treatment | In vivo: 10, 20, 30 mg/kg | In vivo: MCAO rat model | In vivo: Oral gavage | In vivo: 7 d daily p.o. (post-MCAO) | EGCG reduced morphological features of necroptosis. | ^[24]^ |
|  | EGCG | Post-stroke treatment | In vivo: 20 mg/kg | In vivo: MCAO rat model | In vivo: i.p. | In vivo: Single i.p. bolus, post-MCAO | EGCG decreased neurological score, protected neurons, inhibited apoptosis, reduced oxidative stress, increased Bcl‑2, decreased Bax and caspase‑3. | ^[25]^ |
|  | EGCG | Pretreatment | In vitro: 0, 50, 100, 150, 200 µM | In vitro: PC12 cells treated with H₂O₂ | In vitro: Dissolved in culture medium | In vitro:2 h pre-incubation (pre-H₂O₂, washed) | EGCG protected against oxidative stress‑induced apoptosis via PI3K/Akt, GSK-3, cytochrome c and caspase‑3 pathways. | ^[26]^ |
|  | EGCG | Pretreatment | In vivo: 50 mg/kg In vitro: 0, 20, 40 µM | *In vivo:* MCAO rat model In vitro: HT22 cells treated with 5 mM glutamate | In vivo: i.p. In vitro: Dissolved in culture medium | In vivo: Single i.p. bolus pre-MCAO  In vitro: 1 h pre + 24 h co-incubation | EGCG regulated hippocalcin and apoptosis‑related proteins. | ^[27]^ |
|  | EGCG | Pretreatment | In vitro: 10, 20, 30, 40 µM | In vitro: PC12 cells treated with MGO | In vitro: Dissolved in culture medium | In vitro: 3 h pre + 24 h co-incubation | EGCG alleviated MGO‑induced neurotoxicity via MAPK pathway and inhibition of AGEs. | ^[28]^ |
|  | EGCG | Post-stroke treatment | In vivo: 5 µL | In vivo: MCAO/R rat model (2 hours ischemia/6-24 hours reperfusion ) | In vivo: Intracerebroventricular injection | In vivo: Single intracerebroventricular bolus, 0 h post-reperfusion | EGCG inhibited ER stress and improved neurological status via calpain‑mediated TRPC6 proteolysis inhibition and CREB activation through MEK/ERK pathway. | ^[29]^ |
|  | EGCG | Pretreatment | In vivo: 50 mg/kg  In vitro: 1‑50 µM | In vivo: SAH mouse model  In vitro: PC12 cells treated with OxyHb | In vivo: Oral gavage  In vitro: Dissolved in culture medium | In vivo: 14 d daily p.o. (pre-SAH)  In vitro: 30 min pre + 48 h co-incubation | EGCG inhibited Ca²⁺ influx via VGCC, protected mitochondrial function, and reversed autophagy changes (Atg5 decrease, LC3B/Becn‑1 accumulation). | ^[30]^ |
|  | PEG‑PLGA EGCG nanoparticles (EGCG‑NPs) | Pretreatment | In vivo: 75 mg/kg/day | In vivo: SAH mouse model | In vivo: i.p. | In vivo: 7 d daily i.p. (pre-SAH) | EGCG‑NPs inhibited ferroptosis and ferritinophagy after SAH by suppressing HO-1‑mediated iron metabolism reprogramming. | ^[31]^ |
|  | GTPP (EGCG, ECG, EGC, EC) | Pretreatment | In vitro: 0.05‑5 µg/ml | In vitro: PC12 cells treated with OGD/R(3 hours OGD/18-20 hours reperfusion) | In vitro: Dissolved in culture medium | In vitro: 2 d pre-incubation (pre-OGD) | GTPP stimulated 67LR and induced NADPH oxidase‑dependent ROS generation, activating PKCε and preconditioning against OGD/R‑induced cell death. | ^[32]^ |
|  | Catechin | Post-insult treatment | In vitro: 10, 50, 100 µM | In vitro: Hypoxia/reperfusion treatment in microglial cells | In vitro: Dissolved in culture medium | In vitro: 6 h hypoxia + 24 h reperfusion (total 30 h co-exposure) | Catechin up‑regulated Akt and mTOR phosphorylation and inhibited autophagy. | ^[33]^ |
| Preserving BBB integrity and mitochondrial function | EGCG | Post-stroke treatment | In vivo: 50 mg/kg | In vivo: Global cerebral ischemia mouse model | In vivo: i.p. | In vivo: Single i.p. bolus, 0 h post-reperfusion | EGCG inhibited MMP‑9 activation and reduced delayed neuronal death. | ^[34]^ |
|  | EGCG | Post-stroke treatment | In vivo: 50 mg/kg | In vivo: MCAO/R mouse model (80 min ischemia/24 hours reperfusion ) | In vivo: i.p. | In vivo:Single i.p. bolus, 0 h post-reperfusion | EGCG reduced MMP‑9 up‑regulation and neuronal damage. | ^[35]^ |
|  | EGCG | pretreatment | In vitro: 5, 10, 20 µM | In vitro: Primary rat cerebellar granule neurons | In vitro: Dissolved in culture medium | In vitro: 24 h co-treatment | EGCG accumulated in mitochondria and showed selective anti‑apoptotic effects against mitochondrial oxidative stress. | ^[36]^ |
|  | EGCG | Pretreatment | In vivo: 50 mg/kg | In vivo: HI rat model | In vivo: i.p. | In vivo: 4 boluses (2 pre-HI + 2 post-HI, 72 h span) | EGCG increased endothelial and neuronal NOS expression, preserved mitochondrial complexes I‑V and citrate synthase activity. | ^[37]^ |
|  | EGCG | Pretreatment | In vivo: 12.5, 25, 50 mg/kg In vitro: 10 µmol/L | In vivo: MCAO/R rat model (2 hours ischemia/24 hours reperfusion ) In vitro: Co‑culture of rat hippocampal neurons and astrocytes treated with OGD/R | In vivo: i.p. In vitro: Dissolved in culture medium | In vivo: 3 d daily i.p. (pre-MCAO)  In vitro: 4 h OGD co-exposure | EGCG restored NKA activity and enhanced membrane GLT‑1 expression via NKA‑GLT‑1 interaction, ameliorating I/R injury. | ^[38]^ |
|  | EGCG | Post-stroke treatment | In vivo: 3, 9 mg/kg | In vivo: A rat cerebral ischemia-reperfusion model induced by CA/CPR(cardiac arrest/cardiopulmonary resuscitation) | - | In vivo: Single i.v. bolus, 1 min post-Return of spontaneous circulation | EGCG alleviated global cerebral I/R injury by improving mitochondrial dysfunction and regulating ERK pathway. | ^[39]^ |
|  | Catechin | Pretreatment | In vivo: 30, 50 mg/kg | In vivo: Bilateral common carotid artery occlusion rat model (60 min ischemia/30min reperfusion ) | In vivo: i.p. | In vivo: 7 d daily i.p. (pre-ischemia) | Catechin reduced MDA concentration in brain and serum. | ^[40]^ |
|  | Green tea extract (GTE) | Post-stroke treatment | In vitro: 0.1 mg/ml (1.58% catechin, 3.43% EC, 4.64% GCG, 7.8% ECG, 22.7% EGCG) | In vitro: C6 glial treated with OGD/R | In vitro: Dissolved in culture medium | In vitro: 90 min post-OGD (added at reoxygenation) | GTE protected against glial swelling and prevented loss of mitochondrial membrane potential. | ^[41]^ |
| Angiogenesis and neurogenesis | EGCG | Pretreatment | In vivo: 50 mg/kg | In vivo: MCAO rat model | In vivo: i.p. | In vivo: Single i.p. bolus pre-MCAO | EGCG attenuated up‑regulation of PLPP and HSP60 proteins. | ^[42]^ |
|  | EGCG | Pretreatment | In vivo: 50 mg/kg | In vivo: MCAO mouse model | In vivo: i.p. | In vivo: 7 d daily i.p. (starting 0 h post-reperfusion) | EGCG promoted angiogenesis in early stage of ischemic stroke, possibly via Nrf2 up‑regulation. | ^[43]^ |
|  | EGCG | Pretreatment | In vivo: 50 mg/kg In vitro: 50 µM | In vivo: SAH mouse model In vitro: PC12 cells | In vivo: i.p. In vitro: Dissolved in medium | In vivo: 14 d daily p.o. (pre-SAH) In vitro: 30 min pre + OxyHb co-exposure | EGCG promoted neurite outgrowth after SAH via ROS/integrin β1/FAK/p38 pathway. | ^[44]^ |
|  | EGCG | Mixed | In vivo: 7.5, 15 mg/kg In vitro: 25, 50, 100 µM | In vivo: MCAO/R rat model (2 hours ischemia/24 hours reperfusion ) In vitro: Primary rat hippocampal/cortical neurons treated with OGD | In vivo: intravenous injection In vitro: Dissolved in culture medium | In vivo: 2 boluses (30 min pre-MCAO + 0 h post-R) In vitro: 24 h pre + 4 h OGD | EGCG improved synaptic transmission efficiency by regulating excitatory/inhibitory amino acid balance. | ^[45]^ |
|  | ECG/EGCG | Pretreatment | In vitro: 0.5, 1, 2, 4 µM | In vitro: Human brain microvascular endothelial cells (HBMVECs) treated with OGD/R | In vitro: Dissolved in culture medium | In vitro: Pre-hypoxia, co-through OGD/R | ECG/EGCG affected angiogenesis, promoted proliferation, tube formation, migration, inhibited apoptosis and autophagy by regulating VEGF, Bcl‑2, Bax, LC3B, caspase‑3, mTOR, Beclin‑1. | ^[46]^ |
|  | GTPP or EGCG | Pretreatment | In vitro: GTPP 0.1 µg/ml, EGCG 0.5 µM | In vitro: PC12‑TrkB cells treated with H₂O₂ | In vitro: Dissolved in culture medium | In vitro:3 d co-treatment with BDNF | EGCG acted via 67LR and synergized with other GTPP polyphenols to enhance BDNF‑induced neurite outgrowth. | ^[47]^ |
|  | GTPP (EGCG, ECG, EGC, EC) | Pretreatment | In vitro: GTPP 0.1 µg/ml (EGCG 1 µM) | In vitro: PC12 cells | In vitro: Dissolved in culture medium | In vitro: 3 d co-treatment with NGF | GTPP increased mRNA and protein of neurofilament L and GAP‑43; EGCG enhanced NGF‑induced neurite outgrowth and activated ERK. | ^[48]^ |
|  | (-)-Epicatechin‑3‑gallate (ECG) | Pretreatment | In vitro: 0.1, 1, 10 µM | In vitro: C6 cell lineage | In vitro: Dissolved in culture medium | In vitro:1 h pre + 24 h co-incubation (CoCl₂) | ECG improved glutamate uptake and promoted S100B secretion in C6 cells, suggesting astrocyte‑mediated neuroprotection. | ^[49]^ |
| i.p. = intraperitoneal; i.v.= intravenous; p.o.=per os | | | | | | | | |

[1]	UCHIDA S, OZAKI M, AKASHI T, et al. Effects of (-)-epigallocatechin-3-O-gallate (green tea tannin) on the life span of stroke-prone spontaneously hypertensive rats [J]. Clin Exp Pharmacol Physiol Suppl, 1995, 22(1): S302-3.

[2]	SRIVIDHYA R, KALAISELVI P. Neuroprotective potential of epigallo catechin-3-gallate in PC-12 cells [J]. Neurochem Res, 2013, 38(3): 486-93.

[3]	NAGAI K, JIANG M H, HADA J, et al. (-)-Epigallocatechin gallate protects against NO stress-induced neuronal damage after ischemia by acting as an anti-oxidant [J]. Brain Res, 2002, 956(2): 319-22.

[4]	SHAH Z A, LI R C, AHMAD A S, et al. The flavanol (-)-epicatechin prevents stroke damage through the Nrf2/HO1 pathway [J]. J Cereb Blood Flow Metab, 2010, 30(12): 1951-61.

[5]	JIANG C, ZHUGE X, LI D, et al. Epicatechin-mediated modulation of the Nrf2/HO-1 pathway alleviates senile cerebral ischemic/reperfusion injury [J]. Food Sci Nutr, 2024, 12(9): 6521-33.

[6]	LAN X, HAN X, LI Q, et al. (-)-Epicatechin, a Natural Flavonoid Compound, Protects Astrocytes Against Hemoglobin Toxicity via Nrf2 and AP-1 Signaling Pathways [J]. Mol Neurobiol, 2017, 54(10): 7898-907.

[7]	HAN J, WANG M, JING X, et al. (-)-Epigallocatechin gallate protects against cerebral ischemia-induced oxidative stress via Nrf2/ARE signaling [J]. Neurochem Res, 2014, 39(7): 1292-9.

[8]	FU Y, KOO M W. EGCG protects HT-22 cells against glutamate-induced oxidative stress [J]. Neurotox Res, 2006, 10(1): 23-30.

[9]	CHANG C F, CHO S, WANG J. (-)-Epicatechin protects hemorrhagic brain via synergistic Nrf2 pathways [J]. Ann Clin Transl Neurol, 2014, 1(4): 258-71.

[10]	LEE H, BAE J H, LEE S R. Protective effect of green tea polyphenol EGCG against neuronal damage and brain edema after unilateral cerebral ischemia in gerbils [J]. J Neurosci Res, 2004, 77(6): 892-900.

[11]	ZHANG F, LI N, JIANG L, et al. Neuroprotective Effects of (-)-Epigallocatechin-3-Gallate Against Focal Cerebral Ischemia/Reperfusion Injury in Rats Through Attenuation of Inflammation [J]. Neurochem Res, 2015, 40(8): 1691-8.

[12]	HE F, YE B, CHEN J, et al. Effect of EGCG on inflammatory reaction in rats suffered cerebral ischemia/reperfusion injury [J]. Zhong Nan Da Xue Xue Bao Yi Xue Ban, 2021, 46(12): 1325-31.

[13]	ASHAFAQ M, RAZA S S, KHAN M M, et al. Catechin hydrate ameliorates redox imbalance and limits inflammatory response in focal cerebral ischemia [J]. Neurochem Res, 2012, 37(8): 1747-60.

[14]	ZHONG X, LIU M, YAO W, et al. Epigallocatechin-3-Gallate Attenuates Microglial Inflammation and Neurotoxicity by Suppressing the Activation of Canonical and Noncanonical Inflammasome via TLR4/NF-κB Pathway [J]. Mol Nutr Food Res, 2019, 63(21): e1801230.

[15]	KIM S R, SEONG K J, KIM W J, et al. Epigallocatechin Gallate Protects against Hypoxia-Induced Inflammation in Microglia via NF-κB Suppression and Nrf-2/HO-1 Activation [J]. Int J Mol Sci, 2022, 23(7).

[16]	MACHIN A, DIVAMILLENIA D, FATIMAH N, et al. The Effect of Green Tea with EGCG Active Compound in Enhancing the Expression of M2 Microglia Marker (CD206) [J]. Neurol India, 2022, 70(2): 530-4.

[17]	ZHANG J C, XU H, YUAN Y, et al. Delayed Treatment with Green Tea Polyphenol EGCG Promotes Neurogenesis After Ischemic Stroke in Adult Mice [J]. Mol Neurobiol, 2017, 54(5): 3652-64.

[18]	QU Z, MENG F, ZHOU H, et al. NitroDIGE analysis reveals inhibition of protein S-nitrosylation by epigallocatechin gallates in lipopolysaccharide-stimulated microglial cells [J]. J Neuroinflammation, 2014, 11: 17.

[19]	REZNICHENKO L, AMIT T, YOUDIM M B, et al. Green tea polyphenol (-)-epigallocatechin-3-gallate induces neurorescue of long-term serum-deprived PC12 cells and promotes neurite outgrowth [J]. J Neurochem, 2005, 93(5): 1157-67.

[20]	PARK D J, KANG J B, SHAH M A, et al. Epigallocatechin Gallate Alleviates Down-Regulation of Thioredoxin in Ischemic Brain Damage and Glutamate-Exposed Neuron [J]. Neurochem Res, 2021, 46(11): 3035-49.

[21]	JUNG J Y, HAN C R, JEONG Y J, et al. Epigallocatechin gallate inhibits nitric oxide-induced apoptosis in rat PC12 cells [J]. Neurosci Lett, 2007, 411(3): 222-7.

[22]	JUNG J Y, MO H C, YANG K H, et al. Inhibition by epigallocatechin gallate of CoCl2-induced apoptosis in rat PC12 cells [J]. Life Sci, 2007, 80(15): 1355-63.

[23]	PARK D J, KANG J B, KOH P O. Epigallocatechin gallate alleviates neuronal cell damage against focal cerebral ischemia in rats [J]. J Vet Med Sci, 2020, 82(5): 639-45.

[24]	MACHIN A, SYAHARANI R, SUSILO I, et al. The effect of Camellia sinensis (green tea) with its active compound EGCG on neuronal cell necroptosis in Rattus norvegicus middle cerebral artery occlusion (MCAO) model [J]. J Basic Clin Physiol Pharmacol, 2021, 32(4): 527-31.

[25]	NAN W, ZHONGHANG X, KEYAN C, et al. Epigallocatechin-3-Gallate Reduces Neuronal Apoptosis in Rats after Middle Cerebral Artery Occlusion Injury via PI3K/AKT/eNOS Signaling Pathway [J]. Biomed Res Int, 2018, 2018: 6473580.

[26]	KOH S H, KIM S H, KWON H, et al. Epigallocatechin gallate protects nerve growth factor differentiated PC12 cells from oxidative-radical-stress-induced apoptosis through its effect on phosphoinositide 3-kinase/Akt and glycogen synthase kinase-3 [J]. Brain Res Mol Brain Res, 2003, 118(1-2): 72-81.

[27]	PARK D J, KANG J B, KOH P O. Epigallocatechin gallate improves neuronal damage in animal model of ischemic stroke and glutamate-exposed neurons via modulation of hippocalcin expression [J]. PLoS One, 2024, 19(3): e0299042.

[28]	HE Y, YANG Z, PI J, et al. EGCG attenuates the neurotoxicity of methylglyoxal via regulating MAPK and the downstream signaling pathways and inhibiting advanced glycation end products formation [J]. Food Chem, 2022, 384: 132358.

[29]	YAO C, ZHANG J, LIU G, et al. Neuroprotection by (-)-epigallocatechin-3-gallate in a rat model of stroke is mediated through inhibition of endoplasmic reticulum stress [J]. Mol Med Rep, 2014, 9(1): 69-76.

[30]	CHEN Y, HUANG L, ZHANG H, et al. Reduction in Autophagy by (-)-Epigallocatechin-3-Gallate (EGCG): a Potential Mechanism of Prevention of Mitochondrial Dysfunction After Subarachnoid Hemorrhage [J]. Mol Neurobiol, 2017, 54(1): 392-405.

[31]	HUANG L, WANG X, ZHENG Y, et al. EGCG-NPs inhibition HO-1-mediated reprogram iron metabolism against ferroptosis after subarachnoid hemorrhage [J]. Redox Biol, 2024, 70: 103075.

[32]	GUNDIMEDA U, MCNEILL T H, ELHIANI A A, et al. Green tea polyphenols precondition against cell death induced by oxygen-glucose deprivation via stimulation of laminin receptor, generation of reactive oxygen species, and activation of protein kinase Cε [J]. J Biol Chem, 2012, 287(41): 34694-708.

[33]	CHEN C M, WU C T, YANG T H, et al. Green Tea Catechin Prevents Hypoxia/Reperfusion-Evoked Oxidative Stress-Regulated Autophagy-Activated Apoptosis and Cell Death in Microglial Cells [J]. J Agric Food Chem, 2016, 64(20): 4078-85.

[34]	PARK J W, JANG Y H, KIM J M, et al. Green tea polyphenol (-)-epigallocatechin gallate reduces neuronal cell damage and up-regulation of MMP-9 activity in hippocampal CA1 and CA2 areas following transient global cerebral ischemia [J]. J Neurosci Res, 2009, 87(2): 567-75.

[35]	PARK J W, HONG J S, LEE K S, et al. Green tea polyphenol (-)-epigallocatechin gallate reduces matrix metalloproteinase-9 activity following transient focal cerebral ischemia [J]. J Nutr Biochem, 2010, 21(11): 1038-44.

[36]	SCHROEDER E K, KELSEY N A, DOYLE J, et al. Green tea epigallocatechin 3-gallate accumulates in mitochondria and displays a selective antiapoptotic effect against inducers of mitochondrial oxidative stress in neurons [J]. Antioxid Redox Signal, 2009, 11(3): 469-80.

[37]	SUTHERLAND B A, SHAW O M, CLARKSON A N, et al. Neuroprotective effects of (-)-epigallocatechin gallate following hypoxia-ischemia-induced brain damage: novel mechanisms of action [J]. Faseb j, 2005, 19(2): 258-60.

[38]	LIU X X, KE X Y, JIANG C, et al. Na(+)-K(+)-ATPase/GLT-1 interaction participates in EGCG protection against cerebral ischemia-reperfusion injury in rats [J]. Phytomedicine, 2025, 136: 156349.

[39]	QIN S, CHEN M H, FANG W, et al. Cerebral protection of epigallocatechin gallate (EGCG) via preservation of mitochondrial function and ERK inhibition in a rat resuscitation model [J]. Drug Des Devel Ther, 2019, 13: 2759-68.

[40]	FANG F, HAN Y J, CUI Z Q. [The protective effects of tea catechins on the injury of cerebral ischemia and reperfusion in rats] [J]. Zhongguo Zhong Yao Za Zhi, 2001, 26(11): 777-80.

[41]	PANICKAR K S, POLANSKY M M, ANDERSON R A. Green tea polyphenols attenuate glial swelling and mitochondrial dysfunction following oxygen-glucose deprivation in cultures [J]. Nutr Neurosci, 2009, 12(3): 105-13.

[42]	PARK D J, KANG J B, KOH P O. Identification of regulated proteins by epigallocatechin gallate treatment in an ischemic cerebral cortex animal model: a proteomics approach [J]. J Vet Med Sci, 2021, 83(6): 916-26.

[43]	BAI Q, LYU Z, YANG X, et al. Epigallocatechin-3-gallate promotes angiogenesis via up-regulation of Nfr2 signaling pathway in a mouse model of ischemic stroke [J]. Behav Brain Res, 2017, 321: 79-86.

[44]	ZHANG Y, HAN M, SUN X, et al. EGCG Promotes Neurite Outgrowth through the Integrin β1/FAK/p38 Signaling Pathway after Subarachnoid Hemorrhage [J]. Evid Based Complement Alternat Med, 2021, 2021: 8810414.

[45]	DING J, FU G, ZHAO Y, et al. EGCG ameliorates the suppression of long-term potentiation induced by ischemia at the Schaffer collateral-CA1 synapse in the rat [J]. Cell Mol Neurobiol, 2012, 32(2): 267-77.

[46]	FU B, ZENG Q, ZHANG Z, et al. Epicatechin Gallate Protects HBMVECs from Ischemia/Reperfusion Injury through Ameliorating Apoptosis and Autophagy and Promoting Neovascularization [J]. Oxid Med Cell Longev, 2019, 2019: 7824684.

[47]	GUNDIMEDA U, MCNEILL T H, FAN T K, et al. Green tea catechins potentiate the neuritogenic action of brain-derived neurotrophic factor: role of 67-kDa laminin receptor and hydrogen peroxide [J]. Biochem Biophys Res Commun, 2014, 445(1): 218-24.

[48]	GUNDIMEDA U, MCNEILL T H, SCHIFFMAN J E, et al. Green tea polyphenols potentiate the action of nerve growth factor to induce neuritogenesis: possible role of reactive oxygen species [J]. J Neurosci Res, 2010, 88(16): 3644-55.

[49]	ABIB R T, QUINCOZES-SANTOS A, NARDIN P, et al. Epicatechin gallate increases glutamate uptake and S100B secretion in C6 cell lineage [J]. Mol Cell Biochem, 2008, 310(1-2): 153-8.
